# Submerged Corridors of Ancient Gene Flow in an Island Amphibian

**DOI:** 10.1111/mec.17742

**Published:** 2025-04-03

**Authors:** Miranda B. Sherlock, Mark Wilkinson, Simon T. Maddock, Ronald A. Nussbaum, Julia J. Day, Jeffrey W. Streicher

**Affiliations:** ^1^ Herpetology Natural History Museum London UK; ^2^ Department of Genetics, Evolution and Environment University College London London UK; ^3^ School of Natural and Environmental Sciences Newcastle University Newcastle Upon Tyne UK; ^4^ Island Biodiversity and Conservation Centre University of Seychelles Mahé Seychelles; ^5^ Museum of Zoology and Department of Ecology and Evolutionary Biology University of Michigan, Ann Arbor Michigan USA

**Keywords:** caecilian, ddRADseq, gene flow, Indian Ocean, island biogeography

## Abstract

Many island archipelagos sit on shallow continental shelves, and during the Pleistocene, these islands were often connected as global sea levels dropped following glaciation. Given a continental shelf only 30–60 m below sea level, the terrestrial biota of the Seychelles Archipelago likely dispersed amongst now isolated islands during the Pleistocene. *Hypogeophis rostratus* is an egg‐laying, direct‐developing caecilian amphibian found on 10 islands in the granitic Seychelles. Despite the seemingly limited dispersal abilities of this salt‐intolerant amphibian, its distribution on multiple islands suggests likely historic dispersal across now submerged continental shelf corridors. We tested for the genetic signature of these historic corridors using fine‐scale genomic data (ddRADseq). We found that genomic clusters often did not correspond to islands in the archipelago and that isolation‐by‐distance patterns were more consistent with gene flow across a continuous landscape than with isolated island populations. Using effective migration surfaces and ancestral range expansion prediction, we found support for contemporary populations originating near the large southern island of Mahé and dispersing to northern islands via the isolated Frégate island, with additional historic migration across the flat expanse of the Seychelles bank. Collectively, our results suggest that biogeographic patterns can retain signals from Pleistocene ‘palaeo‐islands’ and that present‐day islands can be thought of as hosting bottlenecks or transient refugia rather than discrete genetic units. Thus, the signatures of gene flow associated with palaeo‐islands may be stronger than the isolating effects of contemporary islands in terrestrial species distributed on continental shelf islands.

## 1 Introduction

Recent advances in data availability, theory and methods in molecular and computational biology have culminated in a new paradigm for island biogeography (Fernandez‐Palacios et al. 2015; Heaney 2007; Lomolino 2000). This paradigm shift comes in response to the inference of patterns of island biodynamics that fit neither a strict model of equilibrium (MacArthur and Wilson 1963) nor vicariance (Rosen 1978) and has led to more nuanced interpretations that incorporate multiple scenarios. These scenarios include frequency of over‐water dispersal, variation in gene flow amongst islands, in situ diversification, species‐island age relationships, long‐term persistence of island species and the possibility of continental recolonisation by island species (Heaney 2007). Incorporating historic sea level changes, plate tectonics and other geological processes that influence the extent of island landmass has expanded the field of island biogeography even further (Whittaker et al. 2017).

Remote archipelagos present opportunities to study island biodynamics where immigration from the mainland is generally low and where there is a greater likelihood of lineages produced by in situ speciation (Gillespie and Baldwin 2010). Such in situ speciation is more likely on larger islands, due to the greater opportunity for geographic isolation (especially in islands fragmented by sea level highstands) and the possibility that larger areas contain more habitat types (Losos and Schluter 2000). Islands within archipelagos are more likely to be colonised by migrants originating within the archipelago than from the mainland (Whittaker et al. 2017). In groups for which overseas dispersal is likely to be highly limited, such as amphibians, the short‐distance dispersal within archipelagos is likely to be the only significant source of migrants after initial colonisation (Arjona et al. 2018; Bell et al. 2015; Carvalho and Cardoso 2014). The contribution of interisland distance to population‐genetic structure can be indicated by the degree of isolation‐by‐distance (IBD), where high IBD may suggest that historic episodes of fragmentation are less of an impediment to gene flow than the geographic distance between islands (Papadopoulou and Knowles 2015b).

Islands on shallow continental shelves have experienced varying degrees of connectivity related to Quaternary sea level changes (Rijsdijk et al. 2014; Weigelt et al. 2016). As such, islands that are closer in space may have more similar species compositions due to dispersal opportunities afforded by topographic features that are only apparent during sea‐level lowstands. However, it is important to consider that the presence of emergent land alone is not conducive to species dispersal (Rocha, Perera, Silva, et al. 2016). In the Pleistocene Aggregate Island Complex (PAIC) diversification model, cyclical sea level changes are proposed to act as a ‘species‐pump’ that drives divergence (Heaney 1986; Brown and Diesmos 2002). However, the relative contribution of population fragmentation between small islands (when sea levels rise) and secondary contact/hybridisation (when island aggregates form as sea levels drop) to overall diversification varies and is likely to be system‐specific (see Hosner et al. 2014; Papadopoulou and Knowles 2015b).

**FIGURE 1:**
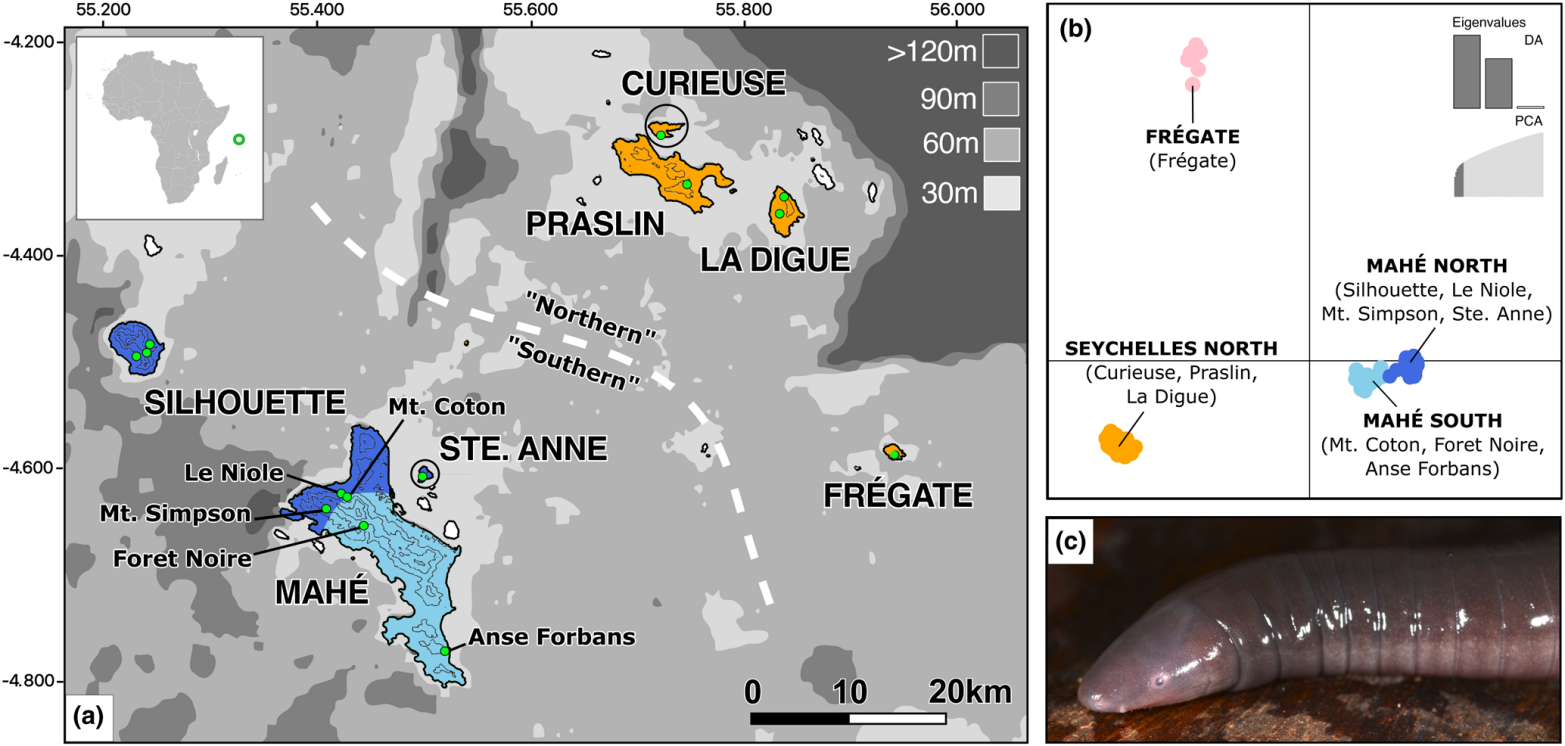
(A) Island connectivity with sea level change. Present‐day emergent land is coloured for cluster membership inferred with DAPC (see B), and islands unsampled in this study are shown in white. Sampling localities are shown by green dots. Elevation (above sea level) is shown by contour lines (200 m spacing). Smaller islands (Ste. Anne and Curieuse) are circled for clarity. The grey shaded area represents exposed land, with the shade of grey in the key indicating metres below current sea level. Bathymetry data from the GEBCO 2024 repository. Inset map shows position of the Seychelles (indicated with a green loop) relative to Africa. Latitude and longitude given in decimal degrees. (B) DAPC of *K* = 4, with eight PCs and two eigenvalues. (C) *Hypogeophis rostratus* , photographed by STM.

The granitic Seychelles comprise a continental fragment that separated from India ca. 63 million years ago (mya) (Collier et al. 2008; Hammond et al. 2013) and is now closer to East Africa (< 1300 km) than India (~2700 km). These islands comprise the exposed elements of ‘Seychellea’ or the Seychelles Microcontinent, which at present is mostly submerged (Starmühlner 1978). The emergent islands are small (total area < 450 km^2^) and are scattered in proximity (< 75 km apart). Drops in sea level of ~50 m will connect all islands, and ~40m will connect all islands except Silhouette (Figure 1A). The most extreme periods of lowstand were centred around the Last Glacial Maximum (LGM) 26.5–19 thousand years ago (kya) (Clark et al. 2009), and prior to this during the penultimate glacial period (PGP) ca. 155–140 kya (Rohling et al. 2017). However, the PGP lowstand was likely not as extreme as during the LGM and may not have connected populations fully or only for a shorter period (Rohling et al. 2017). Major historic highstands of 7.6 ± 1.7 m occurred ca. 125 kya (Dutton et al. 2015), which may have caused range restrictions in the northern islands with more lowland areas. for example, Praslin and La Digue, while leaving the steeper southern islands of Mahé and Silhouette relatively unchanged. These topographical and bathymetric characteristics of the Seychelles offer an opportunity to study historic disconnections and reconnections of island populations and their impacts. Such processes are significant as the isolation of populations can facilitate lineage divergence, while hybridisation that may follow secondary contact can give rise to diversity hotspots (Edwards et al. 2022; Teixeira et al. 2018). While similar disconnection and reconnection processes occur in both continental and insular systems, the identification of marine barriers and sea levels from bathymetry means that in insular systems the historic partitioning of populations—and the size of these partitioned areas—can be inferred with some confidence (Rijsdijk et al. 2014). As such, the properties of islands as separate units can also be applied to disjunct populations in a more continuous landscape (MacArthur and Wilson 1967).

The dispersal of insular amphibians is strongly inhibited by oceanic barriers (Duellman and Trueb 1986; Nussbaum 1984; Vences et al. 2003) such that substantial sea level changes (Oliveira et al. 2016) are expected to impact upon dispersal and genetic population structure. The Seychelles hosts 13 currently recognised endemic amphibian species, several of which have inferred phylogeographies; although to date none have been investigated using genome‐scale data. Previously studied species include the treefrog *Tachycnemis sechellensis* (Maddock, Day, et al. 2014; Nussbaum and Wu 1995), sooglossid frogs (Labisko et al. 2019), and the caecilians *Hypogeophis brevis* and *H. rostratus* (Maddock et al. 2018, 2020). Seychelles caecilians are part of the family Grandisoniidae Lescure, Renous, & Gasc, 1986, relationships within which are well‐studied (Gower et al. 2016, 2011, 2008; Hedges et al. 1993; Loader et al. 2007; Maddock et al. 2016, 2018; Roelants et al. 2007; Sherlock et al. 2024; Wilkinson et al. 2002; Zhang and Wake 2009). The Seychelles radiation of eight caecilian species (seven *Hypogeophis* and the monotypic *Praslinia*) includes both multiple and single island endemics. The multiple island endemic species are all medium‐ or large‐bodied caecilians: *H. rostratus* , *H. alternans* , *H. sechellensis*, *H. larvatus* and *P. cooperi* (Labisko, Griffiths, et al. 2022). In particular, *H. rostratus* is known to occur on 10 islands (Nussbaum 1984) and, relative to many other caecilians, is ecologically versatile, occurring at a range of elevations, habitats and degrees of human disturbance (Maddock et al. 2020). The species has terrestrial eggs and direct development (Brauer 1899, 1879). Previous molecular research using mitochondrial DNA and AFLP loci suggests *H. rostratus* populations diverged into ‘North’ (La Digue, Praslin, Curieuse, Felicite and Frégate) and ‘South’ (Mahé, Ste. Anne, Cerf and Silhouette) island clusters around 1.2 mya, with evidence for hierarchical structure that splits the ‘South’ cluster in Mahé (Maddock et al. 2020). However, morphological data from the same study placed the Frégate population within the ‘South’ group, perhaps due to local‐adaptation overriding the signal of vicariance between the two island groups (Maddock et al. 2020). Fine‐scale genomic data have not been applied to this species despite their potential to overcome issues with low marker resolution by applying much larger numbers of loci (Hohenlohe et al. 2021), and the possibility that they may also provide a clearer resolution of the affinity of Frégate and the extent of gene flow between the ‘South’ and ‘North’ clusters.

Given the broad interisland distribution of *H. rostratus* but presumed restricted overseas dispersal, this species is a good candidate for examining the impacts of cyclical population disconnection and range restrictions on the formation of genetic clusters and the long‐term maintenance of genetic diversity. Among approaches for inferring historical distributions, a theoretically robust but rarely used method involves detecting the origins and axes of range expansions (Peter and Slatkin 2013). In this method, asymmetries in two‐dimensional allele frequency (caused by serial founder events) are used to estimate the spatial origin of an ancestral population prior to range expansion. It has been previously applied to amphibian genome‐scale data (Maier et al. 2019; Waldron et al. 2024) including studies on islands (Buckingham et al. 2022). Measures of genetic diversity (e.g., nucleotide diversity and heterozygosity) can also be used to identify the origin of population expansion (e.g., with the expectation of continuous diversity loss along the axes of expansion, Kimura and Weiss 1964) and to infer historic demographic processes (e.g., bottlenecks associated with a loss of diversity, Hewitt 1996). Understanding the spatial origins of ancestral island populations provides helpful context for interpreting the contemporary distribution of genetic variation across different islands.

We generated fine‐scale population genomic data from multiple island populations of *H. rostratus* using double digest restriction‐site associated (ddRAD) DNA sequencing, which has been successful in identifying fine‐scale genetic structure and recent demographic processes in another insular caecilian (O'Connell et al. 2021). We used these data to (1) characterise fine‐scale genetic structure, (2) investigate patterns of gene flow amongst islands, (3) detect the affinities of clusters and (4) identify plausible dispersal routes of ancestral populations. Given the significant present‐day marine barriers but likely connected historic landscape, our study of a Seychellean amphibian can provide inference on the temporal aspect of genetic structuring, that is, how long it takes for the physical isolation of populations to be reflected in their spatial genetic structure. Based on our results, we addressed the key question: What is a better predictor of observed genomic variation; vicariance associated with contemporary islands or historic dispersal (i.e., gene flow) across now extinct ‘palaeo‐islands’?

## 2 Materials and Methods

### 2.1 Sampling

Liver samples from 90 individuals were included in this study, 84 collected by RAN (UMMZ, University of Michigan) and colleagues during 1985–1991 from Mahé (*n* = 40), Ste. Anne (*n* = 12), Silhouette (*n* = 8), Frégate (*n* = 9), La Digue (*n* = 9), Praslin (*n* = 2) and Curieuse (*n* = 4) and six additional samples from La Digue collected by STM (UCL and NHM) and colleagues in 2014. The populations sampled were selected to be representative of the major granitic islands (Mahé, Praslin, Silhouette and La Digue) and to include some smaller satellite islands (Ste. Anne and Curieuse). Several sites were sampled within Mahé to capture any within‐island spatial genetic structure or variation (Table S1; Figure 1A). Exact sample coordinates were not available for UMMZ samples (field‐code RAN), so sites were chosen for which coordinates could be estimated with reasonable certainty (i.e., nonambiguous, specific named localities). Before combining samples, we checked for differences in heterozygosity and nucleotide diversity (*π*) between the archival (1985–1991) and contemporary (2014) samples. While it is unlikely that there is detectable evolutionary change over 30 years, it is possible that populations that have experienced significant anthropogenic habitat loss (i.e., La Digue, see Senterre 2014) have undergone extreme, recent bottlenecks.

### 2.2 DNA Extraction and Library Preparation

DNA was extracted from tissues with a SeraPure paramagnetic bead method (detailed in Lambert et al. 2019) using a lysis buffer with Proteinase K for tissue digestion. After two rounds of paramagnetic bead clean‐up with 70% ethanol washes, DNA was eluted into 10 mM Tris. Double‐stranded DNA was quantified with a Qubit fluorometer and extraction quality further verified by gel electrophoresis. For each DNA extraction, 200 ng was digested using the restriction enzymes Msp1 (common cutter) and SbfI (rare cutter). The digestions were then suspended on paramagnetic beads and washed with 70% ethanol. The ddRAD library construction method used was modified from Peterson et al. (2012). First, 5′ adapters (6 bp) were ligated to the digestions using T4 DNA Ligase in nine groups of 10, each of which was then pooled. The nine pools were size selected to 438–538 bp using a Blue Pippin (Sage Science) following the protocol of Lambert et al. (2019). The size‐selected samples were then amplified by PCR, using a 5′ labelled forward primer (which was the second index). The PCR products were cleaned (using a bead clean as described above), quantified using a D1000 cartridge on an Agilent TapeStation and pooled. The final library of 90 individuals was sequenced on a HiSeq X by Macrogen (South Korea), with paired‐end sequencing for 150 cycles.

### 2.3 SNP Genotyping and Filtering

FASTQ sequence index files were checked in *FastQC* (http://www.bioinformatics.babraham.ac.uk/projects/fastqc/) and demultiplexed in *Stacks* v. 2.64 (Rochette and Catchen 2017) using the ‘*process_radtags*’ module. Individual FASTQ files were trimmed and filtered in *Trimmomatic* v.0.39 (Bolger et al. 2014) to remove the eight random nucleotides before the SbfI recognition site on forward reads, leaving 137 base pair reads. To optimise the assembly of our data set, we tested multiple clustering thresholds (0.85, 0.9 and 0.95) and calculated the overall heterozygosity, missingness and total number of SNPs genotyped (raw and filtered) and performed a PCA for each of these different thresholds. Reads were then assembled, mapped and SNP genotyped in *dDocent* v.2.94 (Puritz et al. 2014) with paired‐end assembly, a *CD‐HIT* clustering parameter of 0.9, and *BWA* mapping parameters one, three and five for the match score value, mismatch score and gap opening penalty, respectively. We applied a minimum coverage of three for sequences within individuals and six for the minimum number of individuals representing unique sequences. The raw VCF from *dDocent* was filtered using *VCFtools* v.0.1.16 (Danecek et al. 2011). Initial filters applied were a minor allele count of three, a minimum read quality of 30, a minimum number of reads per genotype of three (*‐‐minDP*) and a maximum missing data level (per site) of 0.5 (*‐‐max‐missing*). Secondarily, we filtered for a minor allele frequency of 0.05 and a minimum mean site depth (over all individuals) of 20 (*‐‐min‐meanDP*). Missing data per individual was plotted, and any individuals with more than 60% missing data were removed as a trade‐off between minimising missing data and maximising the number of individuals retained per population; empirical testing indicates that fast‐evolving four‐state sites (i.e., those with A, T, C and G) are only retained in data sets with ≥ 60% missing data (Crotti et al. 2019). Next, the ‘*pop_missing_filter*’ script provided by the authors of *dDocent* was applied to filter by a population‐specific call rate of sites that are missing in more than 20% of individuals in more than two populations. Finally, in‐depth filtering was performed for factors including site depth, allelic balance, strand and paired read representation using the script ‘*dDocent*_filters’ (available at https://github.com/jpuritz/dDocent/raw/master/scripts/dDocent_filters). Discriminant Analysis of Principal Components (DAPC) in *adegenet* v. 2.1.1 (Jombart et al. 2010) and clustering analyses in *structure* (Pritchard et al. 2000) were applied to check for differences in population‐genetic structure recovered at the different filtering thresholds. This analysis also revealed an outlier individual (RAN 31081) which we excluded from further analysis (see Results). Missing data per individual was calculated in *VCFtools*. *Stacks* v. 2.64 was used to convert VCF files into other formats for analysis and to calculate population‐genetic statistics.

### 2.4 Inferring Fine‐Scale Population Coancestry

To infer the number of genetic clusters, we ran Bayesian analysis in *structure* with 100,000 iterations and a burn‐in of 10,000, for a total of 10 independent runs. For this analysis, we thinned SNPs to a single SNP per locus (using vcftools ‘‐‐thin’) leaving 4283 sites. We tested *K* values 1–11, and the optimal *K* value was selected using *Structure Harvester* (Earl and vonHoldt 2012) using the Evanno method (Evanno et al. 2005), and also by considering the mean likelihood of the model. We permuted the outputted clusters for the optimal *K* value using *CLUMPAK* (Kopelman et al. 2015) to address any issues with multimodality and label switching, and plotted results using *distruct* (Rosenberg 2004). To support population‐genetic structural inferences from *structure*, we also ran DAPC in *adegenet*, which uses model selection (via Bayesian Information Criterion or ‘BIC’ values) for sequential *K* values to identify and describe genetic clusters (Jombart et al. 2010). The number of clusters for the DAPC was identified using 80 PCs, and the DAPC was performed with 10 PCs and three eigenvalues. We ran *fineRADstructure* to detect fine‐scale admixture and genetic structuring, and to build a simple tree to infer relationships between clusters using the maximum a posteriori (MAP) state from the highest likelihood MCMC iteration and successively merging populations (Lawson et al. 2012). To assign individuals to clusters, *fineRADstructure* analysis was performed with 100,000 iterations for MCMC burn‐in, 100,000 iterations for MCMC sampling, and a thinning interval of 1000. For tree‐building, default parameters were used.

### 2.5 Isolation‐By‐Distance and Effective Migration Surfaces

We produced a map of the Seychelles (Figure 1A) using information from the GEBCO 2024 gridded bathymetric data set, which for 2024 includes additional source data information from an optical light sensor. To test for isolation‐by‐distance, we ran Mantel tests of geographic (in kilometres) versus chi‐squared and Smouse and Peakall (1999) genetic distances in *GenoDive* v.3.06 (Meirmans 2020) for 20,000 iterations each and using Mantel's *R* as the test statistic. For visualisation purposes, geographic and genetic (chi‐squared) distances were also plotted as a distogram, excluding within population comparisons (to avoid zero geographical distances). To test for spatial heterogeneity in the presence of isolation‐by‐distance, we performed EEMS (estimated effective migration surfaces) analysis in *EEMS* (Petkova et al. 2016). The VCF file was converted to the required ‘diff’ format for EEMS analysis by first converting it to a bed format using the script ‘*vcf2bed*’ (Neph et al. 2012) and then to diff using the program ‘*bed2diffs*’ (distributed with *EEMS*). For EEMS analysis, the total habitat (bounded by coordinate vertices) is divided into ‘demes’ which are triangular grids that migration occurs across. The fineness of the grid is determined by the number of demes: If too few are used then there can be excess smoothing of genetic demes as discrete populations are sampled within a single deme, too many, and spatial uncertainty can arise due to the estimation of parameters in demes without any samples. We tested several deme sizes (100, 200, 500, 700 and 1000) and defined the total habitat as a rectangle enclosing the islands with the corner coordinates (55.1, −4.2), (56.0, −4.2), (56.0, −4.9), (55.1, −4.2). Tuning was performed on the proposal variances of hyperparameters so that proposals were accepted 10%–40% of the time. The hyperparameter ‘*mEffctProposalS2*’ was set to 0.9 following incremental increases, and at this setting the proposals were accepted 42% of the time. Three independent chains were run for the selected deme size for 20 million iterations, checked for convergence, combined and then plotted using the *R* package *reemsplots2* (https://github.com/dipetkov/reemsplots2).

### 2.6 Historical Range Expansion and Migration

Using the *R* package *rangeExpansion* (Peter and Slatkin 2013), we calculated the directionality index (ψ) to perform a spatially explicit ancestral range expansion analysis. We ran the *rangeExpansion* analysis on a NEXUS formatted alignment containing all 77 individuals and 10,874 SNPs with default settings. We ran the analysis with and without the ‘*rworldmap*’ layer, which constrains results to island shape polygons (which has low resolution for the Seychelles and for example does not include Frégate). *RangeExpansion* plots an ‘X’ on the likely origin of expansion and generates a heatmap where yellow indicates a likely origin, red a low likelihood of origin, and grey a negative correlation of diversity and origin from that location. We also conducted a Wilcoxon rank‐sum exact test in *R* v.4.4.1, comparing the expected heterozygosity of Northern (Praslin, Curieuse, La Digue and Frégate) and Southern (Silhouette, Ste. Anne and Mahé) groups.

We ran *TreeMix* (Pickrell and Pritchard 2012) to help distinguish between migration and coancestry in explaining the genetic clusters identified between and within populations. *TreeMix* uses a statistical model to infer the patterns of population splits and mixtures in multiple populations. The model uses population allele frequency data to relate populations to their common ancestor in graph form (a maximum likelihood bifurcating tree that may also include migration edges) and estimates genetic drift using a Gaussian approximation. The migration edges (*m*) are inferred when a purely tree‐like model is a poor fit for the population of interest. For the analysis, an input file was produced using the python script ‘*vcf_to_treemix.py*’ implemented in the *Popgen Pipeline Platform* (https://ppp.readthedocs.io/en/jody/PPP_pages/citations.html). No root was defined as no outgroup was included in the sampling, and jackknife resampling was performed with a block size of 500 to account for possible linkage‐disequilibrium in the data. The analysis was run without sample size correction, as there was only a single individual from the Praslin population. We tested 0–10 migration edges for three runs each (as there was very little improvement in model likelihood after *m* = 6). The number of edges was selected using the Evanno statistic (Evanno et al. 2005) calculated across the three runs in *OptM* (Fitak 2021). Results were plotted using the *R* script ‘*TreeMixPlottingScript.R*’.

## 3 Results

### 3.1 Sequencing and SNP Identification

Across the 90 individuals, a total of 769,133,506 reads were sampled (116.3 Gb, from 28,161,752–207,742,370 reads per library) with 89% of all reads and > 88.3% of reads of all libraries above Q30. For assembly optimisation, the number of SNPs and heterozygosity increased with the *c* value applied (Table S2), but as there was no difference in PCA clustering of samples (Figure S1) we applied a threshold of 0.9 to minimise missingness in our matrix and maximise the number of individuals retained (Table S2). A total of 995,289 sites were genotyped across the 90 individuals. Following initial filtering with a < 60% missing data threshold, 12 individuals were removed, which were from a mixture of PCR libraries and populations, but generally lower quality DNA extractions. Filtering for different population‐specific call rates revealed negligible differences in population structure inferred with DAPC (Figure S3B) and so 20% missing data in any two populations was allowed to increase SNP number (Table S3). After applying preliminary filters for minimum read quality and depth, allele frequency and missingness, 38,039 SNPs and 78 individuals remained. The outlier individual (RAN 31081) was excluded from further analysis as its cluster membership was entirely northern (Figure S2). Given that northern ancestry is very minor in all other Frégate samples in the *K* = 4 *structure* analysis, this northern membership seemed implausible, and it is possible that this is due to sample processing/laboratory error, translocation or dispersal. After removal of the outlier and applying the full filters (using the ‘*dDocent*_filters’ script) the data set contained 10,874 SNPs for 77 individuals with an average missing data of 3.5% (*σ* = 5.1). These 77 individuals were from Mahé (*n* = 34), Ste. Anne (*n* = 10), Silhouette (*n* = 8), Frégate (*n* = 7), La Digue (*n* = 14), Praslin (*n* = 1) and Curieuse (*n* = 3); see Table S1. Comparison of the preliminary (38,039 SNPs) and full‐filter (10,874 SNPs) data sets revealed a lower resolution of population‐genetic structure for the preliminary data set, and so the fully filtered data set was preferred (Figure S4).

### 3.2 Population‐Genetic Structure

Based on the BIC curve (Figure S5), DAPC identified four clusters: (i) ‘Seychelles North’ (La Digue, Curieuse and Praslin), (ii) ‘Frégate’ (Frégate), (iii) ‘Mahé North’ (Silhouette, Ste. Anne, Le Niole and Mt. Simpson) and (iv) ‘Mahé South’ (Anse Forbans, Foret Noire and Mt. Coton). There are no populations with individuals assigned to differing clusters (Table S4). The two Mahé clusters are closest in DAPC space, with the Frégate and Seychelles North clusters more remote (Figure 1B). The Frégate cluster is intermediate with northern and southern clusters on the first axis (Figure 1B).

**FIGURE 2:**
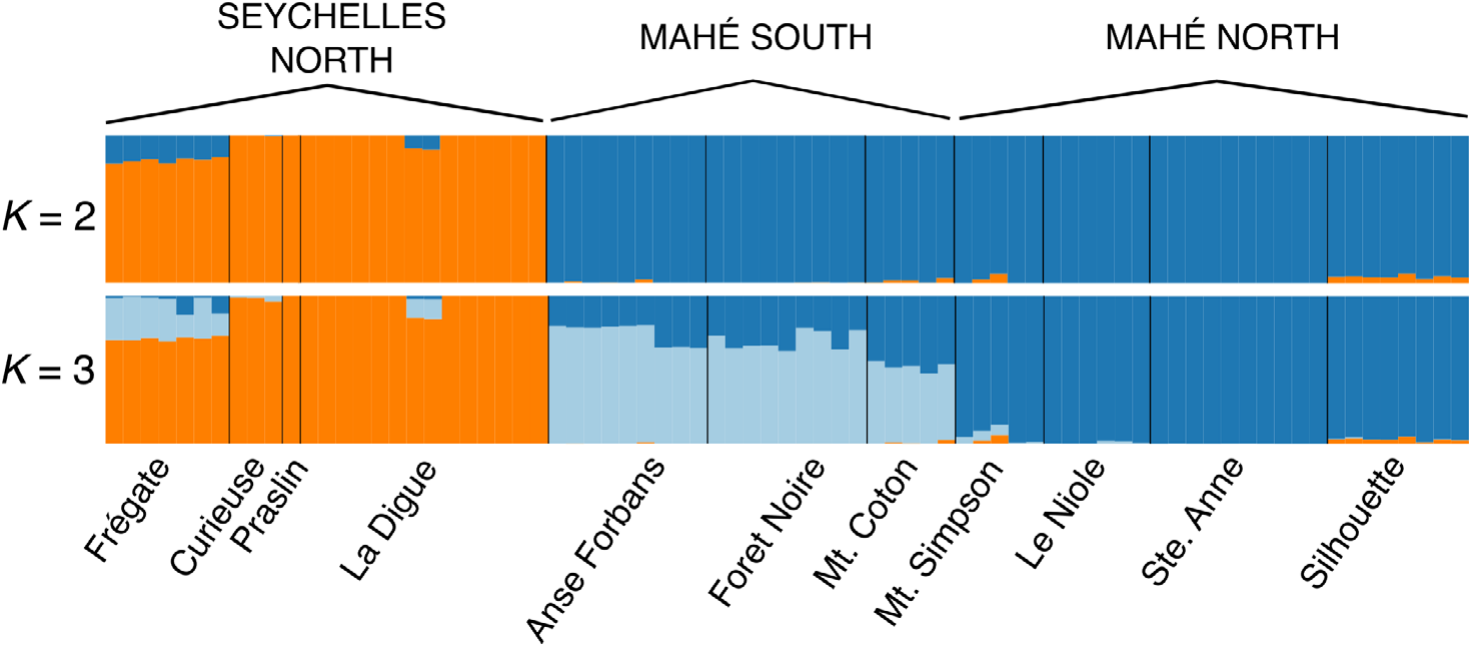
*Structure* plot with 10 runs permuted in *CLUMPAK* and plotted in *distruct* for *K* = 2 and *K* = 3. Vertical bars represent individuals (*n* = 77). Labels above the plots correspond to the major genetic clusters. Colour codes as in (1B).

Using *∆K* values calculated with the Evanno method, *Structure Harvester* selected *K* = 2 as best modelling the observed population structure from the *structure* runs. However, the highest mean likelihood was observed for *K* = 3 that also had some support from *∆K* (Figure 2; Table S5). Both two and three cluster models identify a major split between North (Frégate + La Digue + Praslin + Curieuse) and South (all Mahé populations + Ste. Anne + Silhouette). However, the three cluster model identifies additional structure with ‘South Mahé’ (Anse Forbans + Foret Noire + Mt. Coton), ‘North Mahé’ (Le Niole + Mt. Simpson + Ste. Anne + Silhouette) and ‘Seychelles North’ (Praslin + Curieuse + La Digue + Frégate). The ‘South Mahé’ cluster covers most of the island (excluding the northern tip and Ste. Anne), with admixture at the boundary between the two clusters that decreases into a majority of ‘South Mahé’ membership at the southern extreme of the island. Two La Digue individuals (RAN 31164 and RAN 31165) also contained the ‘South Mahé’ cluster. These individuals do not have high missing data and appear intermediate in PCA of a high‐coverage dataset (minimum within individual coverage of 1000 for a read for use in the reference assembly, Figure S6) which suggests that the mixed membership of these individuals is not due to laboratory error (e.g., sequencing failure or low coverage contamination reads). There were no discernible differences in population‐genetic statistics (Table S6), coancestry (Figure S7), PCA (Figure S6) between the archival and contemporary La Digue samples.

The tree from *fineRADstructure* suggests the presence of two major clusters (‘North’ and ‘South’) with subdivision to a total of four noteworthy clusters (Figure S7). The ‘North’ cluster includes Frégate as the sister population to La Digue, Praslin and Curieuse. The ‘South’ cluster comprises Mahé, Ste. Anne and Silhouette populations. Within the ‘Mahé North’ cluster, Silhouette is adjacent to a cluster comprising Ste. Anne, Le Niole and Mt. Simpson. The coancestry matrix identifies two blocks with high coancestry corresponding to the Mahé South + Mahé North clusters and the Seychelles North + Frégate clusters. The Frégate and Seychelles North groups have very high coancestry, which indicates that they share considerably more ancestry with each other than with the other populations. Relatively low coancestry values in the Mahé South and Mahé North clusters indicate that most of their total coancestry is shared across all clusters (i.e., weak genetic structuring).

### 3.3 Isolation‐By‐Distance, Effective Migration and Spatial Distribution of Genetic Diversity

**FIGURE 3:**
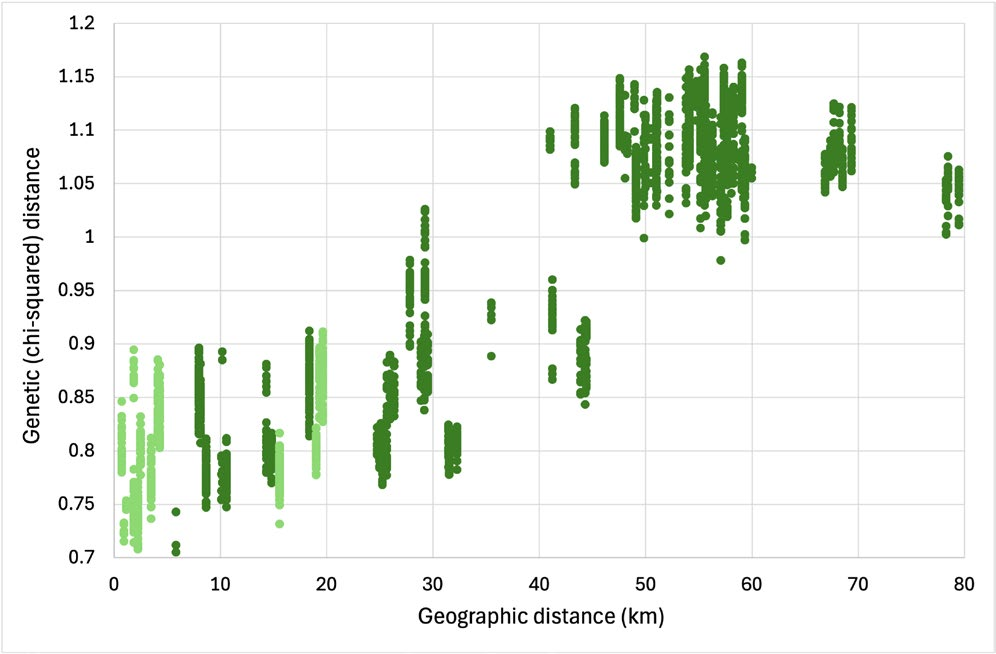
Bivariate plot of pairwise genetic distance (chi‐squared) against pairwise geographical distance (kilometres). Within population distances were not included. Within‐island distances shown in light green, and between‐island shown in dark green.

**FIGURE 4:**
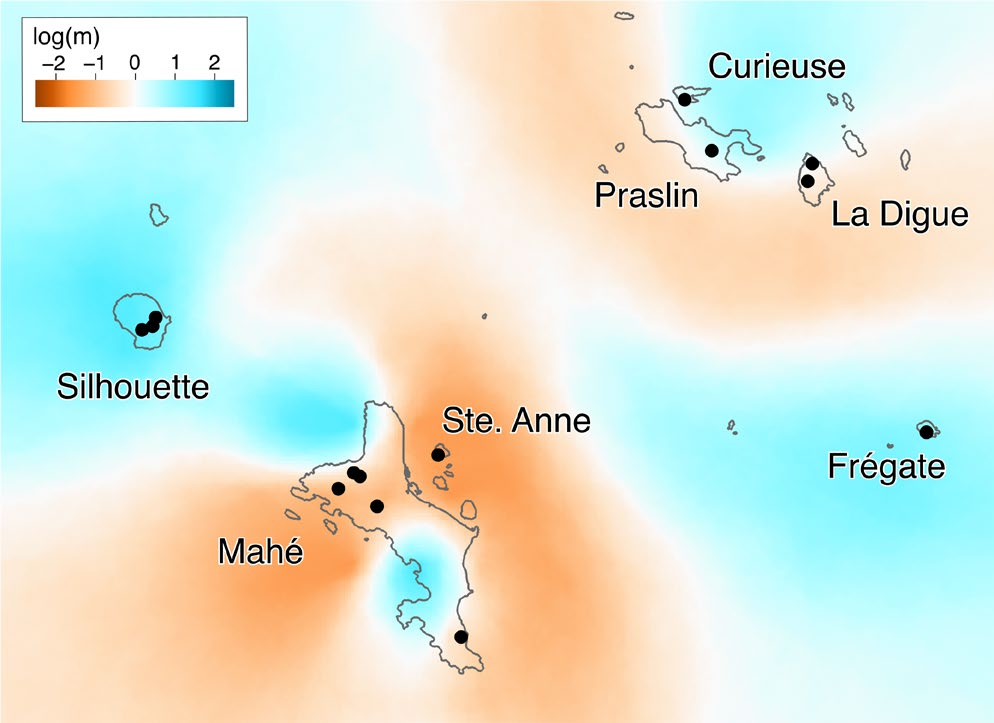
*EEMS* plot of estimated effective migration, with three combined runs of 20 million iterations with 200 demes. Orange represents barriers (to gene flow), and blue corridors (of gene flow) with tone representing the degree. Effective migration rates are given on a log_10_ scale, where migration rates ten times higher than average is log(*m*) = 1 and ten times lower log(*m*) = −1. Sampling localities are indicated by green circles.

The Mantel test of chi‐squared genetic and geographic distances revealed a significant (*p* = 0.05) correlation with an *R* ^2^ of 0.811 (see Figure 3); the test using Smouse & Peakall distances was also significant (*p* = 0.05) with an *R* ^2^ of 0.593. Different deme numbers for analysis in *EEMS* produced concordant results, so 200 demes were selected to reduce the number of demes without sampled populations and the computational burden. The *EEMS* migration plot reveals two major barriers or areas of low gene flow: One separating North Seychelles populations from all other populations and the second dividing Mahé into Mahé North and Mahé South (Figure 4). Conversely, high gene flow is indicated between the islands of Mahé and Silhouette. There is some weak evidence for gene flow between Frégate and Silhouette.

Expected heterozygosity (*H*_E_) is highest in Mahé South populations, followed by Mahé North, Frégate and then Seychelles North. The Wilcoxon rank‐sum exact test found a significant (*p* value = 0.006) difference between the *H*_E_ of Northern and Southern groups (Figure S13). Praslin has the lowest *H*_E_ and highest *π*, but this is unreliable due to the small sample size (*n* = 1). Across all populations, observed heterozygosity (*H*_O_) is more variable but is consistently higher than *H*_E_ (Figure S8). The Mt. Coton and Mt. Simpson populations in the northern montane region of Mahé have the highest *π* (after Praslin) of 0.315 and 0.298, and La Digue has the lowest (0.268). Any geographical pattern of increases or decreases of *π* is less clear than with heterozygosity, although Mahé North and Mahé South populations have generally higher *π* than Seychelles North populations (Figure S8).

### 3.4 Range Expansion and Migration

**FIGURE 5:**
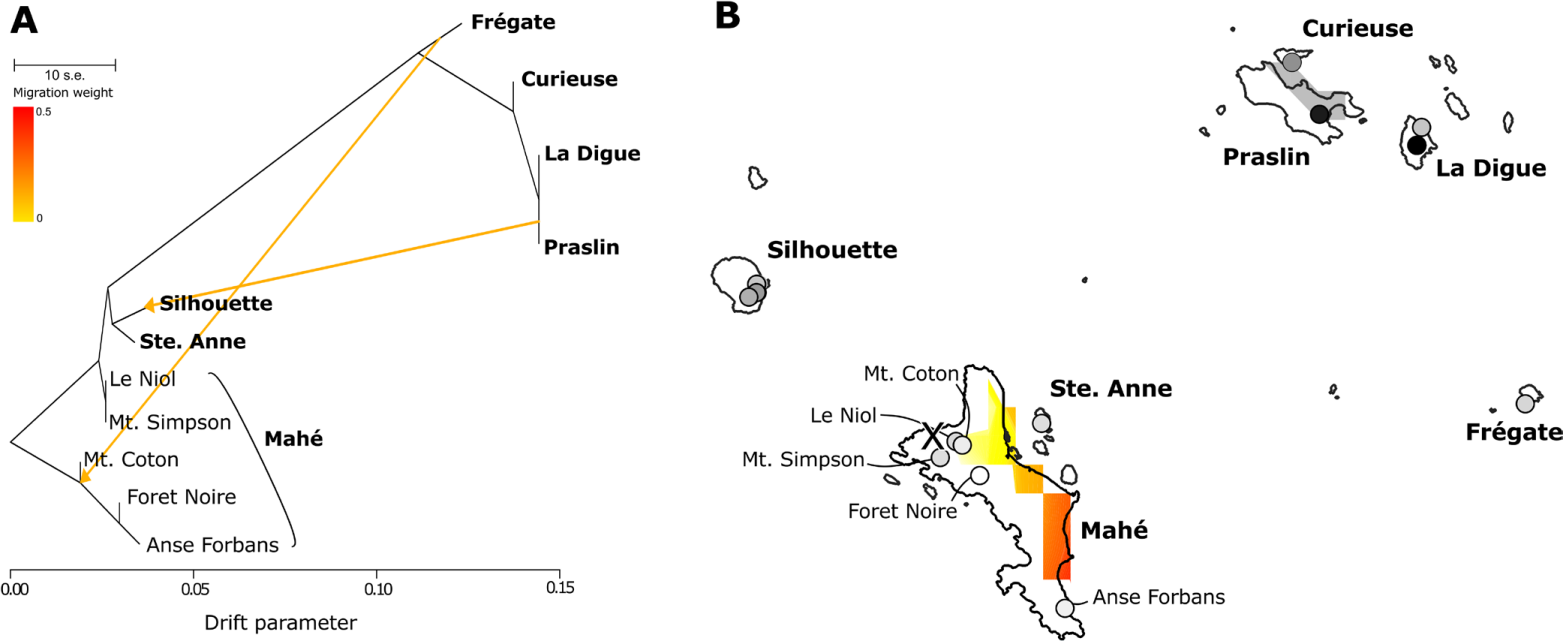
(A) Results from *TreeMix* analysis and (B) results from *rangeExpansion* analysis, islands are labelled in bold and localities within islands (e.g., for Mahé) in regular text. For (A) Migration edges are represented by coloured lines (see inset key for migration weight). For (B) sample localities are plotted as circles, dark shades (black) represent low heterozygosity and light shades (white) high heterozygosity. The heatmap represents likelihood of origin with yellow = high and red = low. The inferred origin of expansion is marked with an ‘X’.

Two edges (*m* = 2) were selected as the best model in *TreeMix* based on the Evanno statistic (Figure S9, Table S7) and the relatively even distribution of errors from the residual plot (Figure S10). The inferred topology (Figure 5A) has a first split between Mahé South (Mt. Coton, Foret Noire and Anse Forbans) and all other populations. The next divergences are of northern Mahé (Le Niol and Mt. Simpson) and all remaining populations, followed by a split between Silhouette and Ste. Anne, and the Seychelles North (Curieuse, La Digue and Praslin) and Frégate populations, with a subsequent split between Frégate and the Seychelles North populations. This analysis identified two migration edges as optimal: from Frégate to Mahé (Mt. Coton) and from Praslin to Silhouette. There are minor differences between the three replicate runs, most notably that run three inferred a migration edge between Praslin + North Mahé rather than Frégate + Mt. Coton (Figure S11), although this run has a less even distribution of error than the other two (Figure S10).

The *rangeExpansion* analysis identified the likely origin of range expansion to be in northern Mahé (between Mt. Simpson, Le Niol and Mt. Coton), in both analyses with and without the *rworldmap* layer (Figures 5B and S12A) and rarefied to a maximum of three samples per locality (Figure S12B). This region also had the highest likelihood of origin (yellow values in the heatmap), followed by southern Mahé and then all other islands.

## 4 Discussion

In addition to influencing island connectivity, topography also determines the extent to which sea level changes cause range expansions and contractions through the increase or reduction of emergent land area (Fernández‐Palacios et al. 2016). For much of the last glacial cycle (120,000 years) sea levels have been significantly lower, resulting in larger and more well‐connected islands (Camoin et al. 2004). Following drastic warming that succeeded the LGM ca. 21 kya, global sea levels have increased by as much as 134 m (Lambeck et al. 2014), which has effectively constrained island biotas into small and isolated refugia. Changes in sea level, therefore, contribute to two major influences of demographic change: migration (through facilitating or inhibiting dispersal between islands) and habitat availability (through permitting expansion or imposing bottlenecks). Long‐term persistence in refugia and secondary contact of isolated lineages can generate diversity hotspots and/or melting pots, which is well established in continental systems (Chiocchio et al. 2021; Dufresnes et al. 2016; Munclinger et al. 2022) but less so in insular ones where dispersal out of refugia may be impeded (but see Mairal et al. 2015; Salvi et al. 2014).

Our population genomic analysis of *H. rostratus* provides new insights into how currently submerged dispersal corridors may contribute to contemporary genetic variation in species living on continental shelf islands. Below we discuss our results for *H. rostratus* in terms of how they relate to patterns observed in the fauna of the Seychelles and other archipelagos and how interisland genomic signatures often do not reflect current distributions, suggesting the maintenance of genetic signatures associated with extinct palaeo‐islands.

### 4.1 Signatures of Northern and Southern Biotic Groups

Results from our *structure* analyses at *K* = 2 (Figure 2) and *fineRADstructure* (Figure S7) identify the primary population structure in *H. rostratus* to be between northern (including Frégate) and southern island populations. This genetic split is common to most phylogeographic studies of other Seychelles taxa (Legrand et al. 2011; Maddock et al. 2020; Nussbaum and Wu 1995; Rocha, Perera, Silva, et al. 2016; Rocha, Perera, Bunbury, et al. 2016; Rocha et al. 2013, 2011; Taylor et al. 2012; Techer et al. 2016; Valente et al. 2014), and because of these biogeographic affinities, the major islands are frequently grouped into ‘North’ (Praslin, La Digue and surrounding islands) and ‘South’ (Silhouette, Mahé and surrounding islands) island groups. This deep divergence reflects that the separation of ‘North’ and ‘South’ by seawater and distance has been a relatively longstanding and significant biogeographic barrier, similar to those caused by mountain ranges in continental systems (e.g., in the Balkan Peninsula, see Thanou et al. 2023). The remote island of Frégate is geographically closer to northern islands (Praslin, Curieuse, La Digue) but, based on bathymetry, has also been connected recently with southern islands (Mahé, Silhouette). The biogeographical affinities of Frégate vary between taxa and traits sampled (e.g., south in *Trachylepis* skinks, Rocha, Perera, Silva et al. 2016 and north in *Hypogeophis rostratus* based on molecules but south based on morphology, Maddock et al. 2020). Previous studies of the Seychelles day geckos, *Phelsuma*, have tried to disentangle if the Frégate population represents an ‘intermediate form’ (Cheke 1981) or a distinct equidistant clade, but this remains unclear (Rocha et al. 2013). For *H. rostratus* , the position of Frégate in the graph of ancestral populations (between northern islands and southern islands) and the migration edge inferred between Frégate and Mahé indicates that the biogeographic position of Frégate may be an intermediate between North and South, as opposed to an isolated outlier (Cheke 1981; Rocha et al. 2013).

Our results from fine‐scale genomic data of *H. rostratus* expand on previous studies with the identification of shared genetic clusters between islands (Mahé and Silhouette) and greater genetic distinctiveness for the remote island of Frégate. Using genomic SNPs, four distinct genetic clusters were identified by DAPC: Seychelles North (Praslin, Curieuse and La Digue), Frégate, South Mahé (Mt. Coton, Foret Noire and Anse Forbans) and North Mahé (Silhouette island, Ste. Anne island, Le Niole and Mt. Simpson). These are not the first exceptions to strict north and south groupings: Others include the Seychelles kestrel *Falco araeus* (Groombridge et al. 2009), *Pelusios* terrapins (Silva et al. 2010), the Seychelles wolf snake *Lycognathophis seychellensis* (Deepak et al. 2021) and the treefrog *Tachycnemis seychellensis* (Maddock, Day, et al. 2014) which lack spatial genetic structure. For *T. seychellensis* and *L. seychellensis* , this was inferred to be due to panmixia at sea level lowstands (Deepak et al. 2021; Maddock, Day, et al. 2014), for *Pelusios* due to recent colonisation (Silva et al. 2010) and for Seychelles kestrels due to study design, as the species is known to be a poor disperser and to have high site fidelity (Groombridge et al. 2009). This variation in observed phylogeographic patterns points toward dispersal ability across and between islands as highly influential in the genetic structuring of Seychelles taxa.

### 4.2 Isolation‐By‐Distance Signal Reflects Historic Larger and More Connected Landscape

Despite the recent postglacial geographic structuring into discrete islands of varying size, the presence of significant isolation‐by‐distance across all populations indicates that the observed genetic structure reflects a larger, more connected habitat for *H. rostratus* . Population‐genetic structure that does not tightly correspond to island allopatry has been reported in other terrestrial vertebrates (e.g., Reynolds et al. 2017; O'Connell et al. 2019), highlighting the general importance of historic dispersal across exposed corridors. The inferred gene flow between Silhouette and northern Mahé, and that these populations comprise a single genetic cluster, suggests that this cluster was formed before the recent separation of the islands by sea level rise. The presence of greater structure within than between islands can occur when there has historically been a larger and more connected ‘palaeo‐island’ (Mairal et al. 2015) or PAIC (Heaney 1986; Brown and Diesmos 2002). In this case, Silhouette + Mahé + Ste. Anne are likely the descendants of a panmictic southern palaeo‐island population. This southern palaeo‐island was partially separated from the ancestors of the northern island populations by distance and a relatively deep trench (> 90 m in parts, see Figure 1A) although this is not deeper than the floor separating the southern islands. As such, our results also corroborate those of Papadopoulou and Knowles (2015b) and Garg et al. (2021), in that bathymetry (i.e., how recently islands were connected) has a major effect on genetic divergence within PAIC. As seen in *Trachylepis* skinks (Rocha, Perera, Silva, et al. 2016) there is evidence for genetic exchange between Silhouette and northern islands (Figures 2, S7 and 5A) although this may have been impeded by the trench that separated these islands in times of less extreme lowstand. We also identified relatively high coancestry of two La Digue samples with southern Mahé populations (Figure S7), although it is possible that the intermediate position of these samples results from relatively recent translocation/dispersal between these islands, with subsequent hybridisation with local individuals. While overseas dispersal of amphibians is considered to occur seldomly, there are some supported instances (see e.g., Bell et al. 2015; Measey et al. 2007; Maddock, Day, et al. 2014; Vences et al. 2003).

The presence of Mahé North, Mahé South and Seychelles North clusters in Frégate in the *K* = 3 *structure* plot (Figure 2), the intermediate position of Frégate on the first DAPC axis (Figure 1B) and the migration edge between Frégate and Mahé inferred by *TreeMix* (Figure A) indicate that despite its northern affinity, the Frégate population retains the signal of historic admixture in a larger, more connected landscape, which further emphasises the role of isolation‐by‐distance in this system. The presence of the South Mahé cluster in Frégate (based on *structure* analyses at *K* = 3) and the higher coancestry values between southern islands and Frégate (as opposed to between southern and northern islands) suggest that the southern islands may have had more significant, recent connections with Frégate than with the northern islands of La Digue, Praslin and Curieuse.

### 4.3 High Genetic Structure and Diversity in Mahé

A genomic study of a flightless Caribbean cricket species emphasised that island size, overall PAIC size and topographic relief were likely to be significant in the propensity to diversify (Papadopoulou and Knowles 2015a). In our study, the largest, most topographically and ecologically diverse island of Mahé comprises two genetic clusters, with barriers identified by EEMS analyses indicating that this structure exceeds that expected from isolation‐by‐distance by more than an order of magnitude (Figure 4). High diversity, both at the population‐ and species‐level, within Mahé has also been identified in several groups including reptiles (*Ailuronyx* bronze geckos; Rocha, Perera, Bunbury, et al. 2016), amphibians (*Tachycnemis* treefrogs; Maddock, Day, et al. 2014, the diminutive *Hypogeophis* caecilian species; Maddock et al. 2018, and Sooglossid frogs; Labisko et al. 2019) and crustaceans (*Seychellum* freshwater crabs; Daniels 2011). Of the islands of the Seychelles bank, Mahé has the greatest continuous extent of montane habitat, which is in stark contrast to the less montane islands of the north and the submerged areas between islands. The location of the transition from North Mahé to South Mahé *structure* cluster membership for *H. rostratus* agrees with that identified in Seychellean freshwater crabs (Daniels 2011), which was suggested to have been caused by limited dispersal due to topographical heterogeneity and regional structuring of drainage basins (Daniels 2011). The north–south transition in cluster membership in *H. rostratus* is similar to the gradient observed in the São Tomé caecilian, inferred to be due to regional environmental variation (O'Connell et al. 2021); however, unlike this study, the north Mahé cluster includes individuals from multiple islands. It is possible that relief also impedes dispersal in *H. rostratus* , which would lead to greater directional genetic structure than expected by isolation‐by‐distance; however, this is somewhat inconsistent with the wide elevational distribution of *H. rostratus* (Maddock et al. 2020).

High structuring can also be caused by geologic processes within islands such as landslides or lava flow, as observed in herbaceous plants of the Canary Islands where populations were likely divided into palaeo‐islands by such processes (Mairal et al. 2015). However, the Seychelles is tectonically stable (Kopp et al. 2009) and in Mahé there have been no reported major landslides or earthquakes. As posited by Maddock et al. (2020) it is possible that this split results from an isolation‐by‐distance signal that is amplified by some kind of other environmental gradient, as was likely for the two São Tomé caecilian *Schistometopum* lineages (O'Connell et al. 2021). However, at present long‐term climatic data is not available, and it is uncertain if island‐scale palaeo‐environmental data can be acquired for the Seychelles (Labisko, Griffiths, et al. 2022). Population‐genetic analysis of the Chinese caecilian *Ichthyophis bannanicus* found that large rivers constitute a major barrier to dispersal (Wang et al. 2015), while a study of Sumatran ranid frogs found no association between diversification patterns and the conformation of palaeodrainage systems (Arifin et al. 2022). Rivers seem unlikely to be a significant barrier for *H. rostratus* . Mahé is covered with a dense network of drainage channels and rivers (Wagle and Hashimi 1990) and field observations indicate that small rivers do not appear to impede the movement of *H. rostratus* (Pawlowski 2016) which actively moves and forages in streams (RAN, MW, STM pers. obs.).

More continuous north–south sampling from different elevations may shed further light on patterns of genetic differentiation within Mahé, although there is little difference in cluster membership between the Foret Noire and Anse Forbans localities either side of the sampling gap, and the transition point between lineages appears to occur in the montane regions around Mt. Coton and Mt. Simpson (Figure 2). Considering that for a large extent of the Pleistocene the islands of Silhouette and Mahé were connected by sea level lowstands, this shared cluster corroborates the suggestion of Maddock et al. (2020) that isolation‐by‐distance is more significant than vicariance. It is possible that in a largely fossorial amphibian with likely limited dispersal (see Stoelting et al. 2014) more significant isolation than recent postglacial sea level rise is required to overwrite the signal of long‐term connectivity (see Papadopoulou and Knowles 2015a).

### 4.4 Historic Range Restrictions May Mask the Origin of Range Expansion

Reconstruction of the relationships of ancestral populations and the origin of range expansion identified the putative ancestral region of *H. rostratus* to have been in northern Mahé (between Mt. Coton and Mt. Simpson). This was likely followed by expansion to the islands of Ste. Anne and Silhouette and eventually (with some lag) to Frégate and the northern islands. In addition, nucleotide diversity is highest in the Mt. Coton and Mt. Simpson populations, and *H*_E_ is significantly higher in Southern than in Northern populations (Figure S12). Such linear gradients of genetic diversity and differentiation (i.e., IBD) indicate continuous dispersal over a large range with few barriers (Kimura and Weiss 1964).

Mahé is mountainous, surrounded by largely flat land that would be emergent with relatively small drops in sea level, and island area has therefore likely been stable historically. Long‐term stability and high genetic diversity support the hypothesis that Mahé hosted the ancestral population (as observed in other PAIC, see Mairal et al. 2015). Alternately, given that Mahé has not been exposed to the same demographic oscillations as smaller, flatter and/or more isolated islands, it may instead have been a refugium for *H. rostratus*. This is where ancestral polymorphism could have been preserved, or a ‘bottleneck’ from which the species may have expanded when sea levels dropped (Bennett and Provan 2008). The lower diversity in northern populations could have resulted from genetic bottlenecks due to range restrictions on the flatter islands, thus erasing any signals of ancestral diversity. The effects of glaciations are likely to be high in the Seychelles, as the islands are surrounded by a flat platform indicating that the range restrictions imposed by sea levels are potentially massive (Fernández‐Palacios et al. 2016). The moderate heterozygote excess in all populations indicates a recent reduction in population size in which allelic richness is lost faster than heterozygosity (Vega et al. 2007). Such a genetic bottleneck supports the existence of larger historic palaeo‐island populations (as in Hawaiian damselflies, see Jones and Jordan 2015), and it is possible that the Northern palaeo‐island is indeed the ancestral area of *H. rostratus* , but that populations have been fragmented by recent sea level rises.

To test these hypotheses further, greater sampling within northern populations would be required, as well as an outgroup taxon to better contextualise the history of population divergences. Further to this, a comparative approach using the sympatric sister species *Hypogeophis alternans* (Sherlock et al. 2024) could help to discern how general the phylogeographic patterns recovered here are. The dispersal ability of Seychellean caecilians is likely influenced by life history (Lourenço et al. 2019) and physiology (Wollenberg et al. 2011); most of these species are distributed on both northern and southern islands, except for the short‐bodied *Hypogeophis* species (which are limited to single islands; Maddock et al. 2017, 2018) and *Praslinia cooperi* (whose large, aquatic larvae makes it highly dependent on freshwater; Nussbaum 1984). In addition, an accurate mutation rate and generation time would allow accurate estimation of divergence times and historic effective population sizes, which would permit explicit investigation of how these demographic changes are influenced by geoenvironmental changes (e.g., glacial cycles). Dating of divergences and range expansions can also be used to explicitly test the hypothesis from Fernández‐Palacios et al. (2016) that there will be maximum extinction during interglacial periods (due to isolation of populations and range restrictions) and maximum immigration during glacial periods (due to emergent connection of islands).

## 5 Conclusions

Studies of other palaeo‐island systems have revealed either genetic correlates with bathymetry data (Papadopoulou and Knowles 2015b; Garg et al. 2021) or lineage‐sharing across islands (Arifin et al. 2022; Oaks et al. 2022; O'Connell et al. 2019; Reynolds et al. 2017; Thanou et al. 2023), collectively supporting the maintenance of genetic signatures associated with now extinct landscapes, even when they are located in close proximity to larger land masses. The findings of our study reveal that current island conformation is not the best predictor of genetic structure in *H. rostratus* . While this finding is somewhat unexpected, given that major genetic divisions are often explained by contemporary geographical features in terrestrial species (O'Connell et al. 2017), it does corroborate early molecular findings that estimates of protein variation better reflect historic patterns of gene flow than contemporary population dynamics (Larson et al. 1984). Results also suggest higher structuring of populations of *H. rostratus* than the north–south divide identified from morphological, mitochondrial and nuclear (AFLP) data (Maddock et al. 2020). High gene flow between Mahé and other islands and barriers to gene flow within Mahé indicated that isolation‐by‐distance and the topology of the landscape are more significant than recent marine vicariance in determining the genetic structure of *H. rostratus* (Daniels 2011). In addition, *H. rostratus* may have dispersed from northern Mahé via the remote island of Frégate, reinforcing the idea that there has been significant dispersal across the Seychelles bank in recent millennia. If high‐resolution genomic data were applied to other Seychelles endemics, it is possible that the infrequent dispersal inferred in other taxa (Rocha et al. 2011) could be revealed to be due to the dominant north–south signal that masks more recent, smaller scale movements as populations are connected and disconnected through periods of sea level change. Determining if and how species deviate from broad biogeographic patterns gives insight into the factors determining the spatial genetic structure of insular species, for example, vagility, reproductive mode, dispersal ability and saltwater tolerance (Arjona et al. 2018; Carvalho and Cardoso 2014; Duellman and Trueb 1986; Kodandaramaiah 2009; Ronce and Clobert 2012). Understanding the relative importance of these factors, and the time scale over which they act, is of vital importance in shrinking and increasingly fragmented habitats. For continental shelf archipelagos at least, it seems that being constrained to postglacial contemporary islands for potentially thousands of generations is not sufficient to overwrite signals of extensive gene flow associated with larger palaeo‐islands.

## Author Contributions

M.B.S.: conceptualisation, writing, laboratory work, data generation, analysis. M.W.: conceptualisation, resource acquisition, supervision, reviewing. J.J.D.: supervision, conceptualisation, reviewing. S.T.M.: resource acquisition, conceptualisation, reviewing. R.A.N.: resources. J.W.S.: conceptualization, writing, laboratory work, supervision, analytical design. M.B.S. wrote the original draft. All other authors contributed to other versions.

## Conflicts of Interest

The authors declare no conflicts of interest.

## Supporting information


Data S1.

## Data Availability

All raw data generated for the study have been uploaded to the NCBI SRA (Accession ID: PRJNA1150784). Input files, alignments and other Supporting Information for this study have been uploaded to the NHM data portal and are available at (https://data.nhm.ac.uk/dataset/submerged‐corridors‐of‐ancient‐gene‐flow‐in‐an‐insular‐caecilian‐amphibian). All tissues are available for requested use at the University of Michigan (UMMZ) and the Natural History Museum, London (NHMUK).
