# Supplementary material for: Submerged Corridors of Ancient Gene Flow in an Island Amphibian
Source: Mol Ecol. 2025 Apr 3;34(9):e17742. doi: 10.1111/mec.17742 (PMC12010468; doi:10.1111/mec.17742)
Supplement: Supplementary file 1 — Data S1. [file MEC-34-e17742-s001.docx]

Supplementary information:

Submerged corridors of ancient gene flow in an island amphibian

## Miranda B. Sherlock, Mark Wilkinson, Simon T. Maddock, Ronald A. Nussbaum, Julia J. Day, Jeffrey W. Streicher

**A**
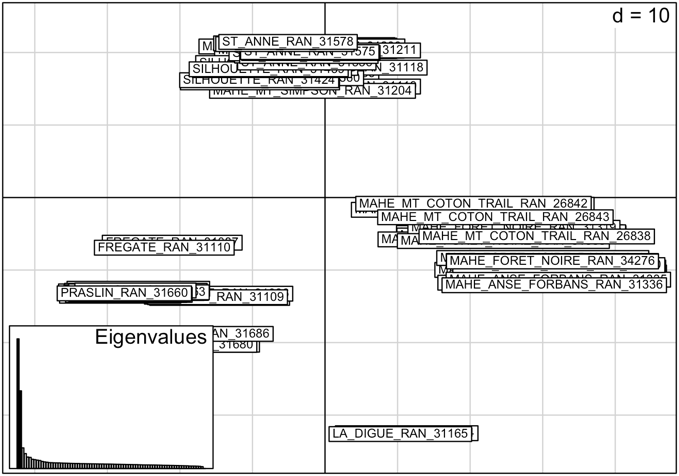
 **B
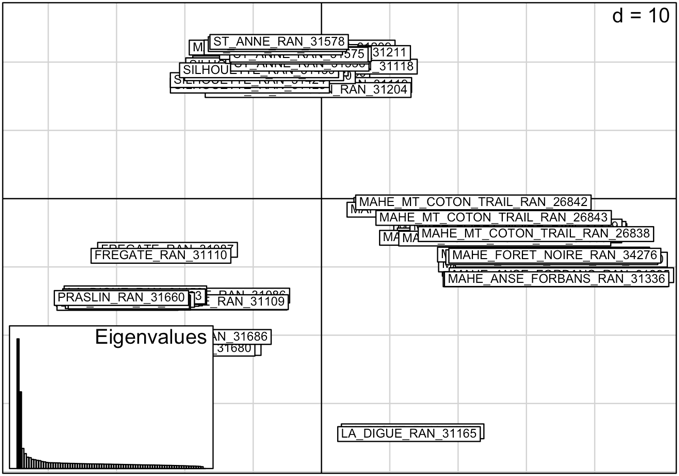
**

**C**
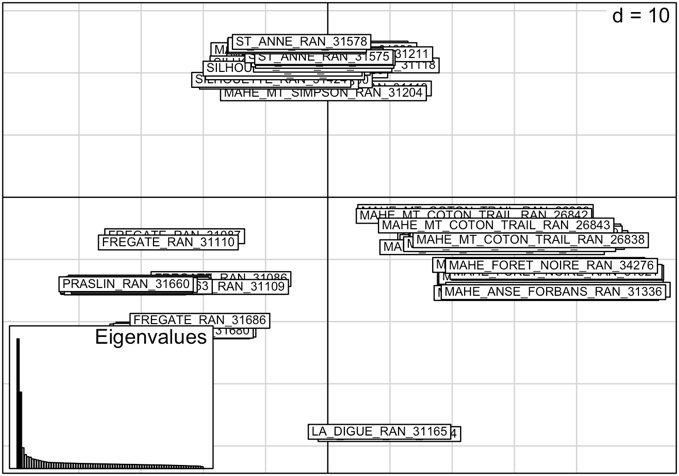


**Figure S1.** PCA of datasets assembled at different cluster similarity (CD-HIT *c* parameter) thresholds, A) 0.85, B) 0.9, C) 0.95


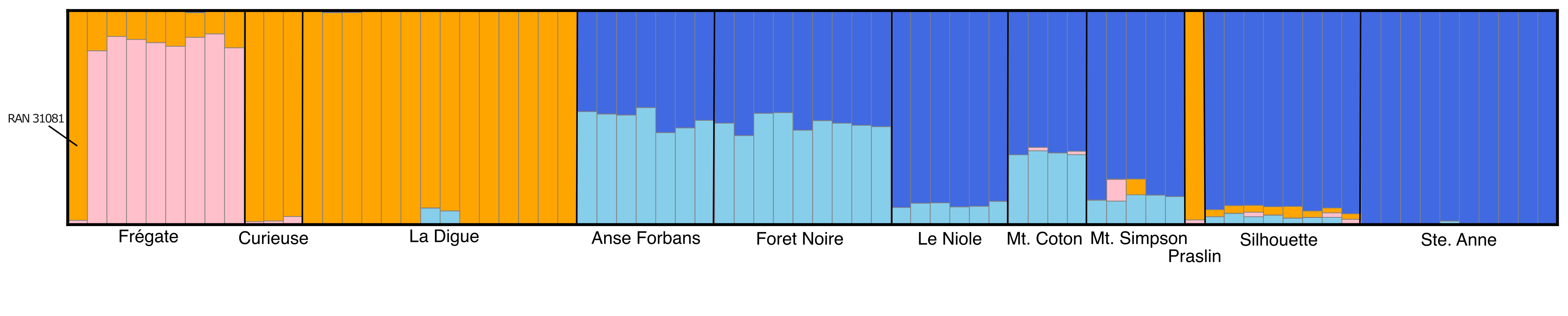


**Figure S2**. *K =* 4 preliminary *structure* plot including the outlier individual RAN 31081 (labelled).

**C**

**D**

**B**

**A**

**Figure S3**. Comparison of population structure inferred at different population specific filtering levels, both with full filtering. For 10% missing data allowed in two populations, A) BIC curve C) and DAPC. For 20% missing data allowed in one population, B) BIC curve, D) and DAPC.


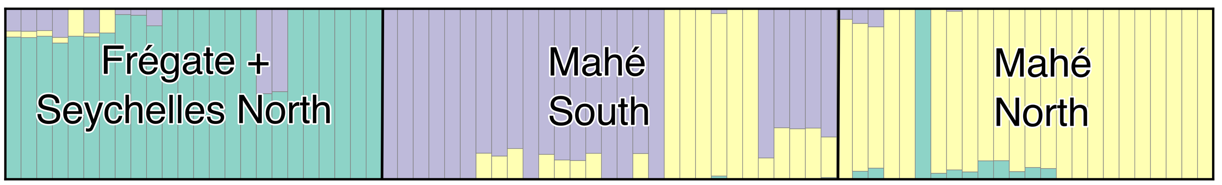

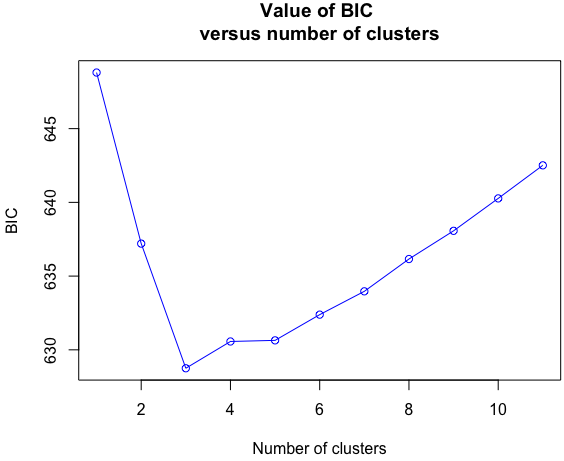


**C**

**B**

**A**


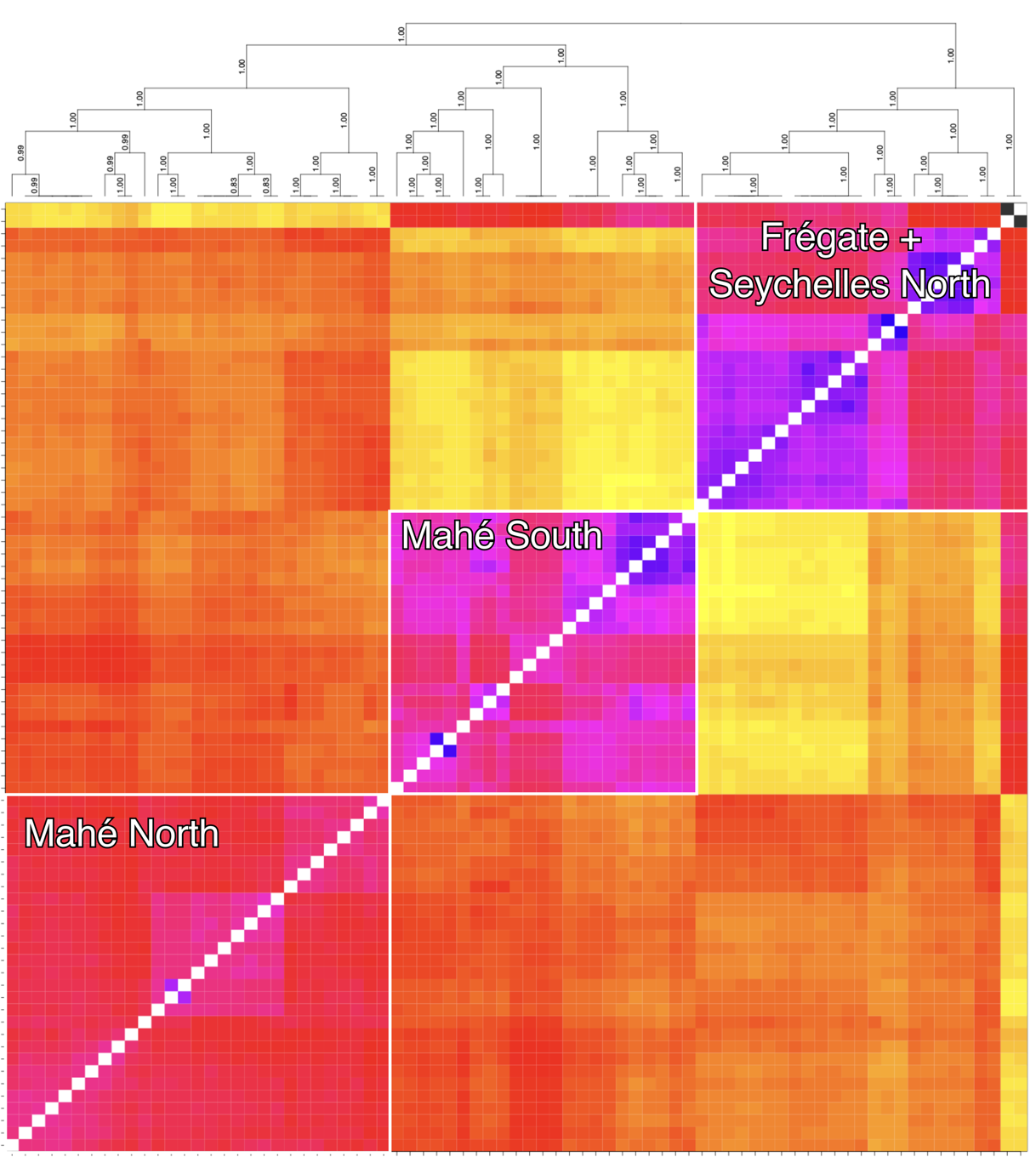


**D**

**Figure S4**. Population genetic structure analysis from preliminary filtering only. For *adegenet*, A) BIC curve, and B) DAPC, colours are as in Figure 2. C) *Structure* plot, where *K* = 3 was selected based on mean log probability and Frégate is grouped within Seychelles North; D) *FineRADstructure*, where white outlines represent major genetic clusters.


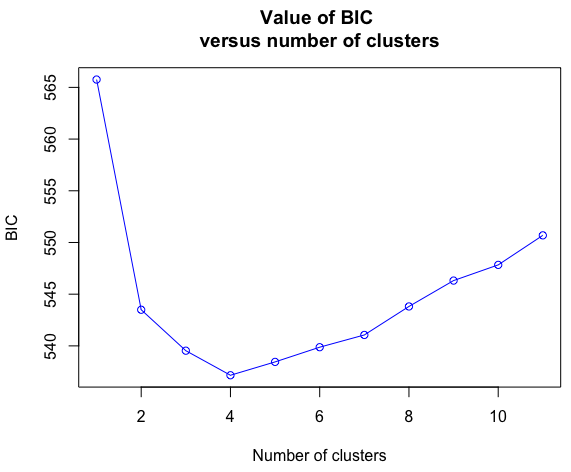


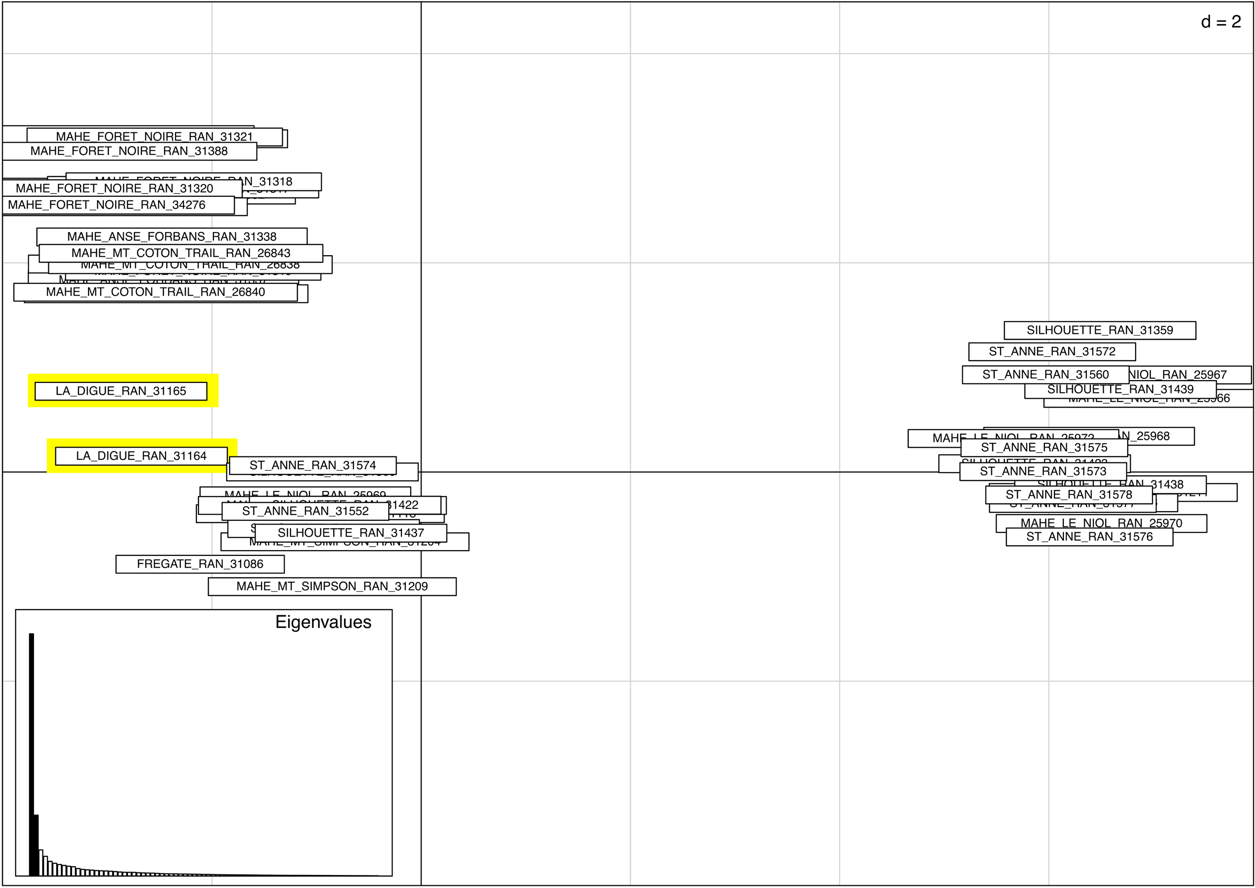
**Figure S5**. Selecting model complexity with DAPC. BIC values plotted against cluster number, showing the ‘elbow’ at *K* = 4.

**Figure S6**. PCA of samples genotyped with a minimum coverage per site of 1,000 (461 sites) showing intermediate position of samples RAN 31164 and RAN 31165, which are highlighted in yellow.


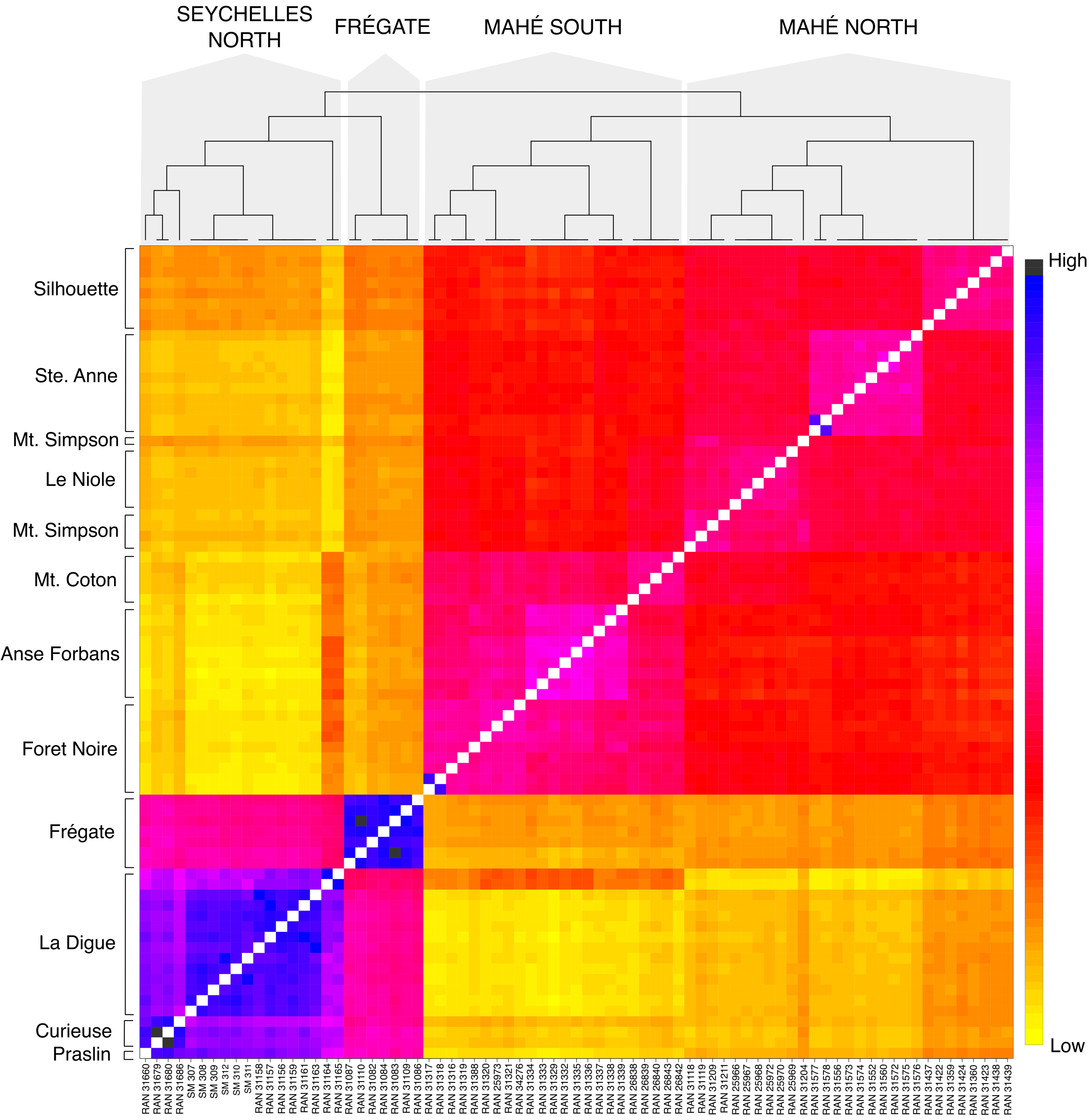


**Figure S7**. Co-ancestry heat map and simple gene tree produced in *fineRADstructure*. The diagonal white line is the null value of the shared co-ancestry within an individual (i.e. self compared with self). Labels on the left indicate the population origin of samples, and on the bottom the individual ID. Darker/colder colours represent higher co-ancestry (i.e. black, blue) and lighter/ warmer colours low co-ancestry (i.e. yellow), see bar on side.


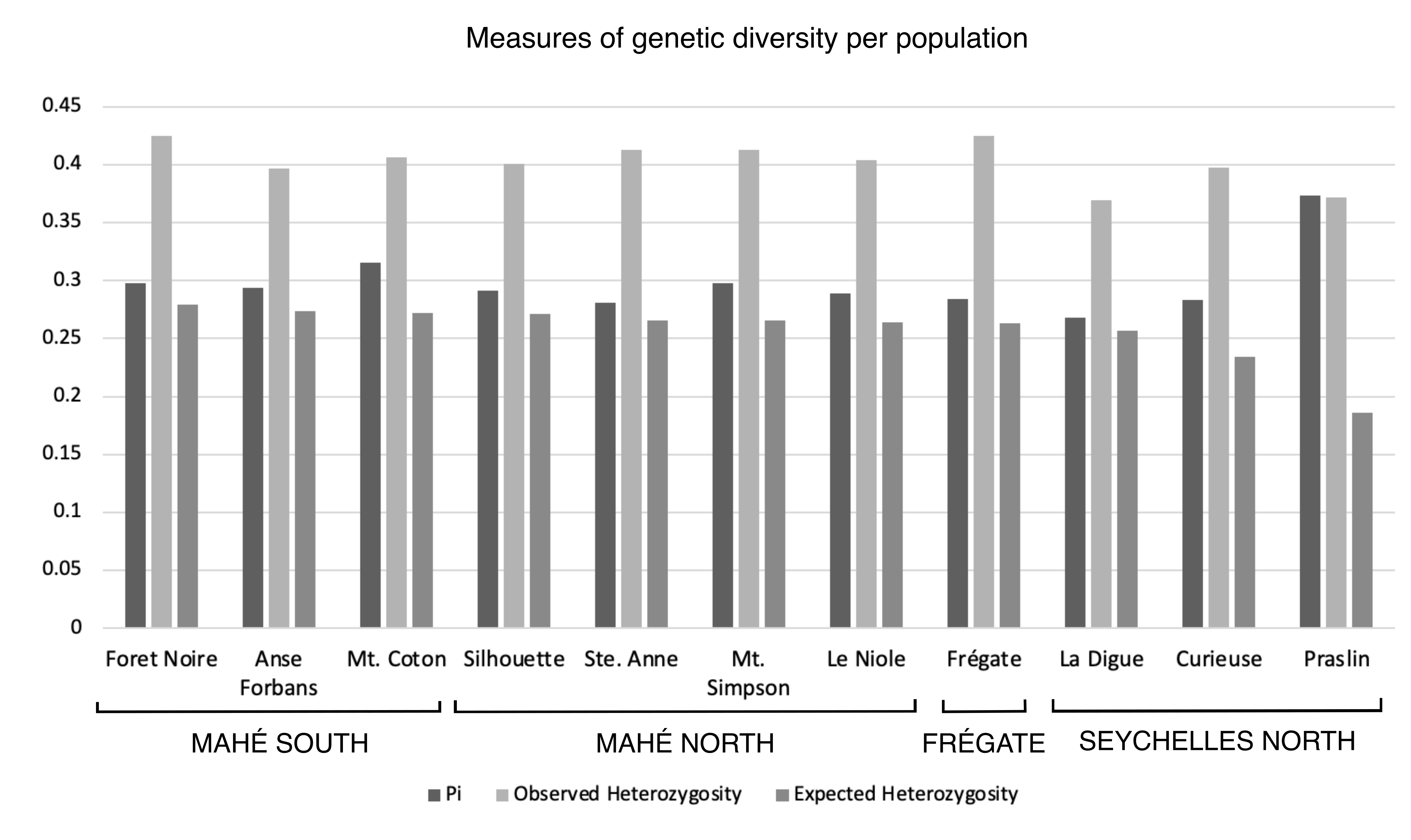


**Figure S8**. Pi and observed and expected heterozygosity per population, calculated in *stacks* using all sites (variant and constant).

**Figure S9**. Results from *OptM* model evaluation using the Evanno method from 10 *TreeMix* runs.

**A
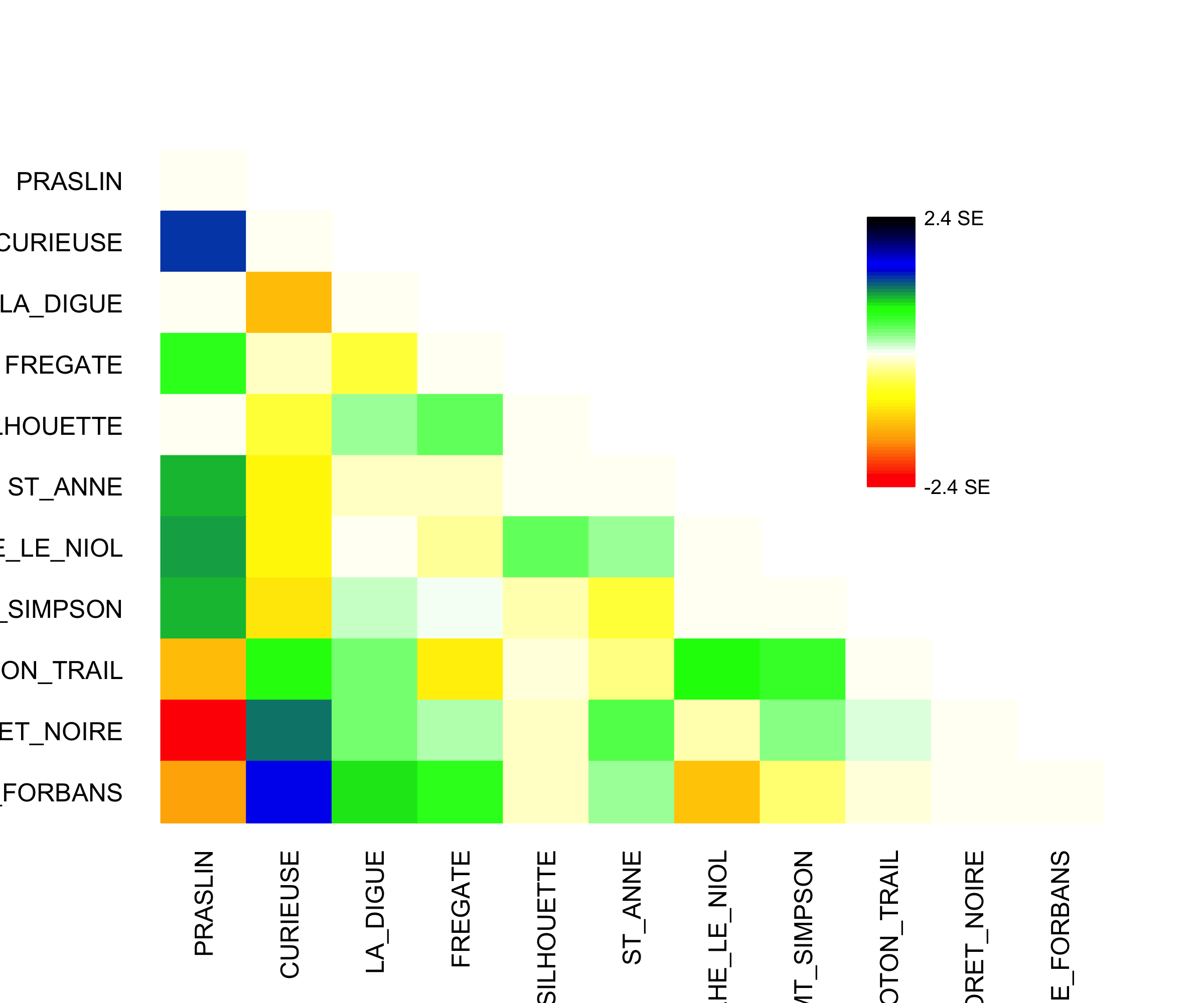
B**

**C**

**Figure S10**. Residual error plots for a single run of the *TreeMix* analysis for the optimum number of hybridisation edges, *m* = 2. A) run 1, B) run 2, C) run 3.

**AB**

**Figure S11**. Alternate trees recovered by the other two *TreeMix* runs at *m* = 2, A) run 2 and B) run 3.

**A B**


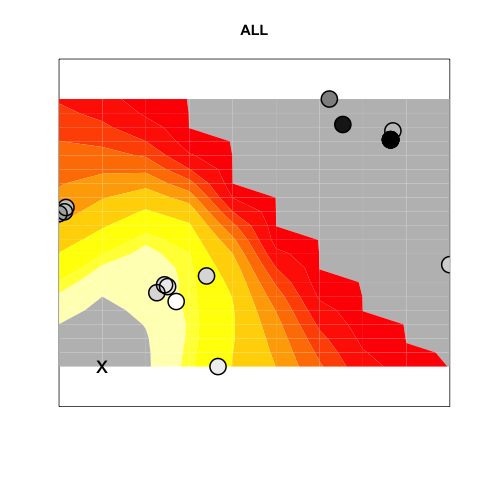


**B**

**A**


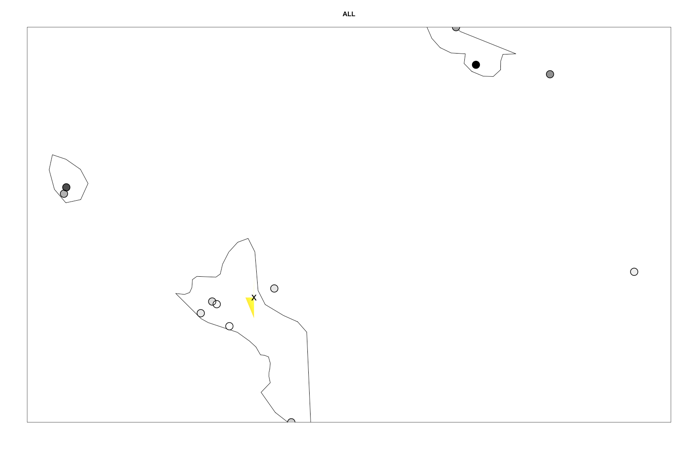


**Figure S12.** A) *RangeExpansion* analysis without the *rworldmap* layer, B) *RangeExpansion* analysis rarefied to maximum of three samples per locality. Sample localities are plotted as circles, dark shades (black) represent low heterozygosity and light shades (white) high heterozygosity. The heatmap represents likelihood of origin with yellow = high and red = low.

**
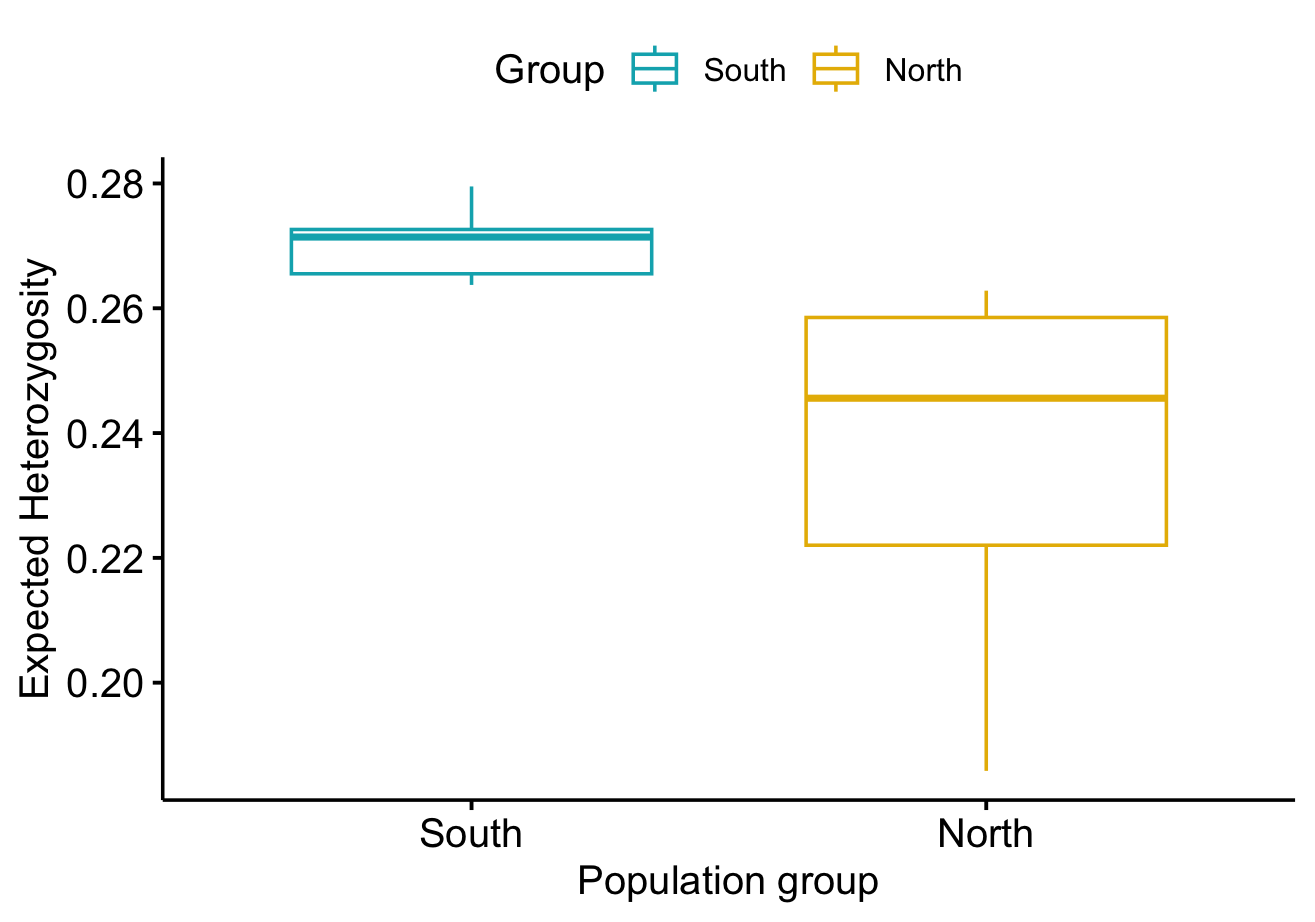
**

**Figure S13.** Results of the non-parametric Wilcoxon rank-sum test, which found a significant (p-value = 0.006) difference between the expected heterozygosity of Northern and Southern groups.

**Table S1.** Sample information. Voucher ID refers to the museum ID of the sample, UMMZ = University of Michigan Museum of Zoology. Sample ID prefixes are RAN (Ronald A. Nussbaum, UMMZ collection) and SM (Simon T. Maddock, BMNH collection).

| Voucher ID | Sample ID | Island | Locality | In final dataset? | Missing data % |
| --- | --- | --- | --- | --- | --- |
| UMMZ 200497 | RAN 31679 | Curieuse | N/A | Y | 0.4 |
| UMMZ 200498 | RAN 31680 | Curieuse | N/A | Y | 0.2 |
| UMMZ 200515 | RAN 31685 | Curieuse | N/A | N | N/A |
| UMMZ 200516 | RAN 31686 | Curieuse | N/A | Y | 8.1 |
| UMMZ | RAN 31081 | Frégate | N/A | N | N/A |
| UMMZ | RAN 31082 | Frégate | N/A | Y | 0.0 |
| UMMZ | RAN 31083 | Frégate | N/A | Y | 0.1 |
| UMMZ | RAN 31084 | Frégate | N/A | Y | 0.2 |
| UMMZ | RAN 31086 | Frégate | N/A | Y | 0.6 |
| UMMZ | RAN 31109 | Frégate | N/A | Y | 0.4 |
| UMMZ | RAN 31110 | Frégate | N/A | Y | 0.5 |
| UMMZ | RAN 31087 | Frégate | N/A | Y | 2.1 |
| UMMZ | RAN 31088 | Frégate | N/A | N | N/A |
| UMMZ | RAN 31155 | La Digue | N/A | N | N/A |
| UMMZ | RAN 31156 | La Digue | N/A | Y | 2.4 |
| UMMZ | RAN 31157 | La Digue | N/A | Y | 0.8 |
| UMMZ | RAN 31158 | La Digue | N/A | Y | 1.0 |
| UMMZ | RAN 31159 | La Digue | N/A | Y | 0.6 |
| UMMZ | RAN 31161 | La Digue | N/A | Y | 0.6 |
| UMMZ | RAN 31163 | La Digue | N/A | Y | 0.4 |
| UMMZ | RAN 31164 | La Digue | N/A | Y | 8.3 |
| UMMZ | RAN 31165 | La Digue | N/A | Y | 6.0 |
| UMMZ 193026 | RAN 31329 | Mahé | Anse Forbans | Y | 11.9 |
| UMMZ 193027 | RAN 31330 | Mahé | Anse Forbans | N | N/A |
| UMMZ 193028 | RAN 31331 | Mahé | Anse Forbans | N | N/A |
| UMMZ 193029 | RAN 31332 | Mahé | Anse Forbans | Y | 4.2 |
| UMMZ 193030 | RAN 31333 | Mahé | Anse Forbans | Y | 6.7 |
| UMMZ 193031 | RAN 31334 | Mahé | Anse Forbans | N | 14.8 |
| UMMZ 193032 | RAN 31335 | Mahé | Anse Forbans | Y | 9.0 |
| UMMZ 193033 | RAN 31336 | Mahé | Anse Forbans | N | 12.4 |
| UMMZ 193034 | RAN 31337 | Mahé | Anse Forbans | Y | 0.2 |
| UMMZ 193035 | RAN 31338 | Mahé | Anse Forbans | Y | 1.1 |
| UMMZ 193036 | RAN 31339 | Mahé | Anse Forbans | Y | 12.9 |
| UMMZ 193037 | RAN 31340 | Mahé | Anse Forbans | N | N/A |
| UMMZ | RAN 31388 | Mahé | Foret Noire | Y | 1.1 |
| UMMZ 192940 | RAN 31315 | Mahé | Foret Noire | N | N/A |
| UMMZ | RAN 31316 | Mahé | Foret Noire | Y | 0.0 |
| UMMZ 192941 | RAN 31317 | Mahé | Foret Noire | Y | 0.0 |
| UMMZ 192942 | RAN 31318 | Mahé | Foret Noire | Y | 0.0 |
| UMMZ 192943 | RAN 31319 | Mahé | Foret Noire | Y | 0.0 |
| UMMZ 192944 | RAN 31320 | Mahé | Foret Noire | Y | 1.7 |
| UMMZ | RAN 31321 | Mahé | Foret Noire | Y | 1.2 |
| UMMZ | RAN 25973 | Mahé | Foret Noire | Y | 14.3 |
| UMMZ 200467 | RAN 31604 | Mahé | Foret Noire | N | N/A |
| UMMZ 200576 | RAN 34276 | Mahé | Foret Noire | Y | 1.1 |
| UMMZ 200008 | RAN 26838 | Mahé | Mt. Coton | Y | 1.7 |
| UMMZ 200009 | RAN 26839 | Mahé | Mt. Coton | Y | 13.7 |
| UMMZ 200010 | RAN 26840 | Mahé | Mt. Coton | Y | 25.0 |
| UMMZ 200012 | RAN 26842 | Mahé | Mt. Coton | N | 19.7 |
| UMMZ 200013 | RAN 26843 | Mahé | Mt. Coton | Y | 7.9 |
| UMMZ | RAN 25966 | Mahé | Le Niole | Y | 0.1 |
| UMMZ | RAN 25967 | Mahé | Le Niole | Y | 0.1 |
| UMMZ | RAN 25968 | Mahé | Le Niole | Y | 0.1 |
| UMMZ | RAN 25969 | Mahé | Le Niole | Y | 8.1 |
| UMMZ | RAN 25970 | Mahé | Le Niole | Y | 0.2 |
| UMMZ | RAN 25972 | Mahé | Le Niole | Y | 0.1 |
| UMMZ | RAN 31209 | Mahé | Mt. Simpson | Y | 0.1 |
| UMMZ | RAN 31211 | Mahé | Mt. Simpson | Y | 0.1 |
| UMMZ | RAN 31118 | Mahé | Mt. Simpson | Y | 2.0 |
| UMMZ | RAN 31119 | Mahé | Mt. Simpson | Y | 9.3 |
| UMMZ | RAN 31120 | Mahé | Mt. Simpson | N | N/A |
| UMMZ | RAN 31204 | Mahé | Mt. Simpson | Y | 4.8 |
| UMMZ 200434 | RAN 31552 | Ste. Anne | N/A | Y | 0.1 |
| UMMZ 200435 | RAN 31554 | Ste. Anne | N/A | N | N/A |
| UMMZ 200436 | RAN 31556 | Ste. Anne | N/A | Y | 0.2 |
| UMMZ 200438 | RAN 31560 | Ste. Anne | N/A | Y | 0.1 |
| UMMZ 200445 | RAN 31572 | Ste. Anne | N/A | Y | 0.1 |
| UMMZ 200446 | RAN 31573 | Ste. Anne | N/A | Y | 0.1 |
| UMMZ 200447 | RAN 31574 | Ste. Anne | N/A | Y | 0.2 |
| UMMZ 200448 | RAN 31575 | Ste. Anne | N/A | Y | 0.2 |
| UMMZ 200449 | RAN 31576 | Ste. Anne | N/A | Y | 0.9 |
| UMMZ 200450 | RAN 31577 | Ste. Anne | N/A | Y | 1.6 |
| UMMZ 200451 | RAN 31578 | Ste. Anne | N/A | Y | 1.2 |
| UMMZ 200452 | RAN 31579 | Ste. Anne | N/A | N | N/A |
| UMMZ 192880 | RAN 31437 | Silhouette | N/A | Y | 1.1 |
| UMMZ 192881 | RAN 31438 | Silhouette | N/A | Y | 2.5 |
| UMMZ 192882 | RAN 31439 | Silhouette | N/A | Y | 3.0 |
| UMMZ 193056 | RAN 31359 | Silhouette | N/A | Y | 0.8 |
| UMMZ 193057 | RAN 31360 | Silhouette | N/A | Y | 1.6 |
| UMMZ 192907 | RAN 31422 | Silhouette | N/A | Y | 0.5 |
| UMMZ 192908 | RAN 31423 | Silhouette | N/A | Y | 1.1 |
| UMMZ 192909 | RAN 31424 | Silhouette | N/A | Y | 0.7 |
| UMMZ 200490 | RAN 31660 | Praslin | N/A | Y | 0.8 |
| UMMZ 200500 | RAN 31756 | Praslin | N/A | N | N/A |
| BMNH 2005.1803 | SM 307 | La Digue | N/A | Y | 3.0 |
| BMNH 2005.1804 | SM 308 | La Digue | N/A | Y | 1.4 |
| BMNH 2005.1805 | SM 309 | La Digue | N/A | Y | 3.2 |
| BMNH 2005.1806 | SM 310 | La Digue | N/A | Y | 10.3 |
| BMNH 2005.1807 | SM 311 | La Digue | N/A | Y | 7.4 |
| BMNH 2005.1808 | SM 312 | La Digue | N/A | Y | 5.3 |

**Table S2**. Results for optimisation of the CD-HIT cluster similarity parameter *c.* ‘Filtered SNPs’ here refers to the preliminary filters detailed in text, but with a genotype call rate of 0.95 applied to all populations. ‘Individuals’ refers to the number of individuals in the filtered dataset, from which *H*_E_ and missingness was calculated.

| ***c* value** | **CD-HIT clusters** | **Individuals** | **Raw SNPs** | **Filtered SNPs** | ***H*_E_** | **Avg. missingness** |
| --- | --- | --- | --- | --- | --- | --- |
| 0.85 | 57,946 | 78 | 930,651 | 40,315 | 0.50 | 1.24% |
| 0.9 | 70,246 | 78 | 995,289 | 45,532 | 0.57 | 1.18% |
| 0.95 | 72,152 | 77 | 1,176,556 | 51,744 | 0.63 | 1.24% |

**Table S3**. Number of SNPs genotyped at different population specific filtering levels.

| Percentage missing data permitted | For how many populations | Number of SNPs genotyped |
| --- | --- | --- |
| 10 | 2 | 6,222 |
| 20 | 1 | 6,540 |
| 20 | 2 | 10,874 |

**Table S4**. Membership of populations to clusters inferred by DAPC.

| Population | Frégate | Mahé South | Mahé North | Seychelles North |
| --- | --- | --- | --- | --- |
| Frégate | 7 | 0 | 0 | 0 |
| Curieuse | 0 | 0 | 0 | 3 |
| Praslin | 0 | 0 | 0 | 1 |
| La Digue | 0 | 0 | 0 | 14 |
| Mahé (Anse Forbans) | 0 | 9 | 0 | 0 |
| Mahé (Foret Noire) | 0 | 9 | 0 | 0 |
| Mahé (Mt. Coton) | 0 | 5 | 0 | 0 |
| Mahé (Le Niole) | 0 | 0 | 6 | 0 |
| Mahé (Mt. Simpson) | 0 | 0 | 5 | 0 |
| Ste. Anne | 0 | 0 | 10 | 0 |
| Silhouette | 0 | 0 | 8 | 0 |

**Table S5.** Results from *structure* model selection using the Evanno method, as applied in *Structure Harvester*.

| *K* | Mean LnP(*K*) | Stdev LnP(*K*) | Δ*K* |
| --- | --- | --- | --- |
| 1 | -321247.690 | 4.820 | N/A |
| 2 | -274848.280 | 9.238 | 4307.992 |
| 3 | -268245.850 | 1645.464 | 27.596571 |
| 4 | -307052.580 | 132232.638 | 0.204510 |
| 5 | -318816.470 | 138280.452 | 4.710593 |
| 6 | -981963.320 | 1423934.501 | 0.924489 |
| 7 | -328698.180 | 94513.092 | 6.758783 |
| 8 | -314226.500 | 76073.287 | 11.915034 |
| 9 | -1206170.600 | 1808821.168 | 0.976865 |
| 10 | -331139.910 | 77812.448 | 18.331226 |
| 11 | -882506.760 | 1859978.242 | N/A |

**Table S6**. Population genetic statistics from the La Digue archival (RAN) and contemporary (SM) samples, calculated in *Stacks*.

| **Collector** | **No. individuals** | **Obs. Het** | **Exp. Het** | **π** |
| --- | --- | --- | --- | --- |
| RAN | 8 | 0.3779 | 0.2576 | 0.2754 |
| SM | 6 | 0.3643 | 0.2383 | 0.2618 |

**Table S7**. Likelihoods for *TreeMix* analyses of different datasets for each *m* value (number of hybridisation edges). Values highlighted in bold are models selected by *OptM* using the Evanno method.

| **Hybridisation edge number (*m*)** | **With Praslin, no sample size correction** | **With Praslin, sample size correction** | **Without Praslin, no sample size correction** | **Without Praslin, sample size correction** |
| --- | --- | --- | --- | --- |
| 0 | 276 | 216 | 197 | 78 |
| 1 | 317 | 280 | **205** | 199 |
| 2 | **308** | **288** | 265 | **268** |
| 3 | 326 | 293 | 273 | 269 |
| 4 | 343 | 294 | 287 | 277 |
| 5 | 333 | 289 | 266 | 264 |
| 6 | 340 | 295 | 274 | 276 |
| 7 | 341 | 288 | 276 | 272 |
| 8 | 350 | 295 | 274 | 273 |
| 9 | 344 | 288 | 277 | 276 |
| 10 | 342 | 294 | 299 | 306 |
